# Satellite tracking resident songbirds in tropical forests

**DOI:** 10.1371/journal.pone.0278641

**Published:** 2022-12-30

**Authors:** Andrew Hart Reeve, Mikkel Willemoes, Luda Paul, Elizah Nagombi, Kasun H. Bodawatta, Troels Eske Ortvad, Gibson Maiah, Knud Andreas Jønsson

**Affiliations:** 1 Natural History Museum of Denmark, University of Copenhagen, Copenhagen, Denmark; 2 New Guinea Binatang Research Centre, Madang, Papua New Guinea; Sichuan University, CHINA

## Abstract

Advances in tracking technology have helped elucidate the movements of the planet’s largest and most mobile species, but these animals do not represent faunal diversity as a whole. Tracking a more diverse array of animal species will enable testing of broad ecological and evolutionary hypotheses and aid conservation efforts. Small and sedentary species of the tropics make up a huge part of earth’s animal diversity and are therefore key to this endeavor. Here, we investigated whether modern satellite tracking is a viable means for measuring the fine-scale movement patterns of such animals. We fitted five-gram solar-powered transmitters to resident songbirds in the rainforests of New Guinea, and analyzed transmission data collected over four years to evaluate movement detection and performance over time. Based upon the distribution of location fixes, and an observed home range shift by one individual, there is excellent potential to detect small movements of a few kilometers. The method also has clear limitations: total transmission periods were often short and punctuated by lapses; precision and accuracy of location fixes was limited and variable between study sites. However, impending reductions in transmitter size and price will alleviate many issues, further expanding options for tracking earth’s faunal diversity.

## Introduction

The world’s animals are continuously on the move as they search for food, breeding opportunities, and territory [1]. Advancing technology has allowed us new insight into the movements of some of the planet’s most charismatic and mobile species [2–4]. However the subject of these studies—terrestrial and marine megafauna [5–7], as well as avian long-distance migrants [8, 9]—represent extreme outliers in the animal kingdom in terms of body size and degree of movement. The greatest part of earth’s faunal diversity is made up of small, tropical, resident species whose movement patterns remain mostly invisible to us.

Tracking technology is steadily becoming smaller and cheaper, which makes it possible to study a more representative swathe of earth’s faunal diversity. This opens exciting new possibilities for testing broad ecological and evolutionary hypotheses [7, 10–13]. Quantifying the fine-scale, long-term movement patterns of animals can answer difficult, longstanding questions in biology, such as whether range limits and community composition are governed by species’ dispersal abilities or by interspecific competition; specific hypothesis testing scenarios are explored in Jønsson et al. [13]. Expansion of tracking studies to include more small, resident tropical species will also be valuable for the conservation of many of earth’s most threatened ecosystems [14]. Detailed movement data can elucidate species’ habitat and territory requirements, and inform reserve planning by showing how habitat gaps, edges and corridors affect animals’ movements and home ranges [15, 16].

Satellite tracking passerine birds in tropical rainforests offers a promising, yet unexplored, avenue to expand the scope of movement studies to smaller, less mobile species. Compared to other terrestrial vertebrate groups, passerines are conspicuous, diverse, and easy to capture. Their ecology and phylogenetic relationships are relatively well studied, and they are frequently used as a model group in evolutionary studies [e.g., 17–19; reviewed in 20]. However, resident passerines push the limits of satellite tracking in terms of small body size and subtlety of movement. Tropical rainforests may also pose a technological challenge. Specific environmental conditions are known to affect transmitter performance [21–23], and structurally dense rainforest canopies may obstruct satellite connections and inhibit solar charging. Radio telemetry is currently the standard method for tracking tropical passerines [e.g., 24–27], but acquiring long-term, high quality data from more than a few individuals is difficult or impossible because of the intensive field effort required. Satellite tracking may provide a low-effort, high-output alternative that makes large-scale tracking of these animals much more feasible.

We used passerine birds in New Guinea to test whether current satellite tracking technology provides a viable means for detecting the movement patterns of small, resident tropical animals. Taking advantage of the island’s rugged, mountainous topography, we worked across several different sites spanning from the lowlands to montane cloud forest. We harnessed thirteen individuals from six species of bowerbird and honeyeater with solar-powered transmitters, and installed stationary transmitters in the lowlands and highlands to serve as controls. We then analyzed four years of transmission data to evaluate how effectively we were able to detect the fine-scale movements of individual birds; and how data transmission varied over time within this system.

## Methods

### Study areas

**Fig 1:**
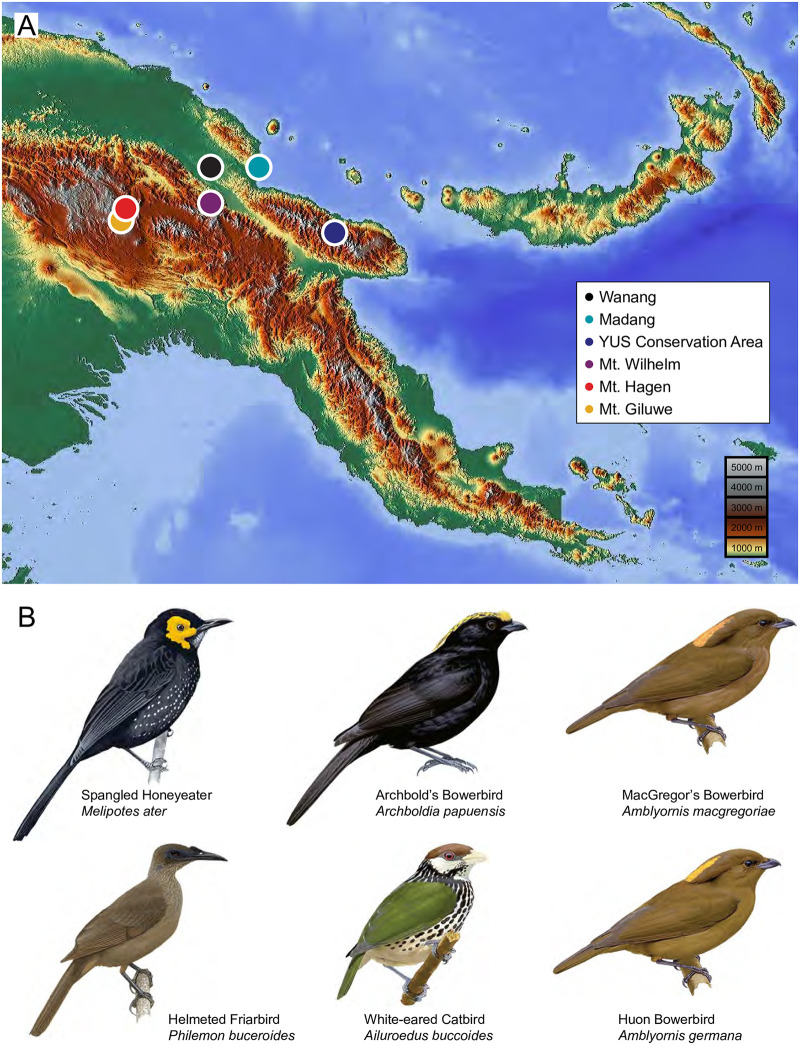
Study areas in Papua New Guinea (A), and tracked bird species (B). Base map and data from OpenStreetMap and OpenStreetMap Foundation. Bird illustrations used with permission from Alada, Barcelona.

New Guinea is the world’s largest tropical island, with varied habitats distributed across a broad, rugged elevational gradient. We conducted our work in Papua New Guinea across three field seasons from 2016–2018. Our study sites across the country (Fig 1A, Table 1) varied in elevation, climate, and habitat, allowing us to evaluate transmitter performance across a range of environmental conditions. Sites covered an elevational span from sea level to 3,000 masl. Forest character varied with elevation, but also in terms of age and intactness. Lowland sites included a small coastal village in heavily degraded mosaic habitat, and the outskirts of a small village located within a very extensive tract of intact lowland forest. Mid-elevation sites contained old secondary forest as well as primary forest with small cleared or regenerating patches. High elevation sites varied from secondary forest on flat ground to pristine cloud forest on steep mountain slopes. We worked at several sites across New Guinea’s dominant central mountain range. These included the northern slopes of Mt. Wilhelm, the tallest mountain in Papua New Guinea, and Mt. Giluwe and Mt. Hagen further to the west. Additional sites were located in the Saruwaged Range of the Huon Peninsula. The mountains of the Huon represent the largest outlying range on the island, hosting several endemics that are absent from the Central Range. Finally, we worked in two lowland sites in the Ramu River basin and along the northern coast.

**Table 1:** Study area details, including year visited, elevation, geographic coordinates, and brief habitat descriptions.

Site	Year	Elev. (masl)	Coordinates	Habitat
YUS, Huon Peninsula	2016	3000	-6.095°, 146.916°	Primary montane forest with small patches of grassland
	2018	1700	-6.161°, 146.840°	Lower montane forest with patchy disturbance
Wanang, Ramu basin	2017	200	-5.228°, 145.173°	Secondary lowland forest and gardens in village outskirts
Mt. Wilhelm	2017	2700	-5.815°, 145.160°	Primary montane forest
	2017	2300	-5.759°, 145.183°	Secondary montane forest bordering village
Mt. Hagen	2018	2900	-5.794°, 143.961°	Primary montane forest
Mt. Giluwe	2018	2700	-5.930°, 143.880°	Montane forest; presumed old secondary growth
	2018	2600	-5.928°, 143.861°	Secondary montane forest and forested garden land
Nagada	2018	0	-5.156°, 145.795°	Coastal village

### Species selection

Candidate species for tracking were chosen from a pool of Papua New Guinean passerine birds meeting a few key criteria. Minimum body mass was 100 g, so that the 5 g transmitters did not exceed 5% of the individuals’ body mass. Strictly ground-dwelling species were avoided, as the solar-powered transmitters need some exposure to direct sunlight to function. Our aim was to test how well we could detect the movements of sedentary birds within their home ranges, and also if we could detect small dispersals beyond these home ranges. We therefore chose resident species that were expected to be largely sedentary, but that we suspected potentially made regular or sporadic short-range movements, based on published findings or our own experience. Six species of bowerbird (Ptilonorhynchidae) and honeyeater (Meliphagidae) fit these criteria (Fig 1B).

Bowerbirds are famous for their complex and spectacular courtship behavior. Adult males build and maintain bowers during much of the year, an activity that demands high site fidelity [28]. We tracked four bowerbird taxa: MacGregor’s Bowerbird *Amblyornis macgregoriae macgregoriae* (2 females, 3 males), Huon Bowerbird *Amblyornis germana* (2 females), Archbold’s Bowerbird *Archboldia papuensis sanfordi* (2 males), and White-eared Catbird *Ailuroedus buccoides geislerorum* (1 unsexed individual). Three of these species inhabit the interior of montane forest, while the catbird occurs mostly below 800 masl in lowland and hill forest [29]. Breeding males of MacGregor’s Bowerbird maintain bowers from roughly May to February [28], but females and immature males may move to lower elevations in the non-breeding season [30]. Huon Bowerbird closely resembles MacGregor’s Bowerbird, which is its sister species [19]. Though it has a different bower construction, it has only recently been split from MacGregor’s Bowerbird [29, 31], and therefore comparatively little is known about its ecology. Archbold’s Bowerbird is a patchily distributed and generally scarce montane species [28]. Little is known about its movement. It occupies the monotypic genus *Archboldia*, and has a unique appearance and bower construction, but a recent molecular analysis [19] demonstrated that it is embedded within *Amblyornis* along with MacGregor’s and Huon Bowerbirds. White-eared Catbird belongs to the bowerbird genus *Ailuroedus*, which contains pair-breeding species that do not build bowers.

We also tracked two honeyeater species: Helmeted Friarbird *Philemon buceroides novaeguineae* (1 male) and Spangled Honeyeater *Melipotes ater* (2 unsexed individuals). Helmeted Friarbird is a lowland species that favors disturbed habitat [31], and is the only species in the study not endemic to New Guinea. It is a successful colonizer, with a broad range spanning Australia, Wallacea, and New Guinea and nearby islands. Small local movements have been observed in certain parts of its range [32]. Spangled Honeyeater, by contrast, is restricted to the interior of montane forest on the Huon Peninsula and has not colonized any of the other mountain ranges in New Guinea. The comparison can provide insight as to whether a lineage’s colonization success is related to the extent of the movements of individuals [13]. Thus, our expectation is that Helmeted Friarbirds are more mobile, with larger home ranges.

Tracked birds were captured and released in appropriate breeding habitat for the respective species. All these sites were located within extensive tracts of similar habitat. Thus, all birds had good possibilities to disperse at least a few kilometers in some directions without meeting significant barriers of unsuitable habitats or elevations.

### Tracking

**Fig 2:**
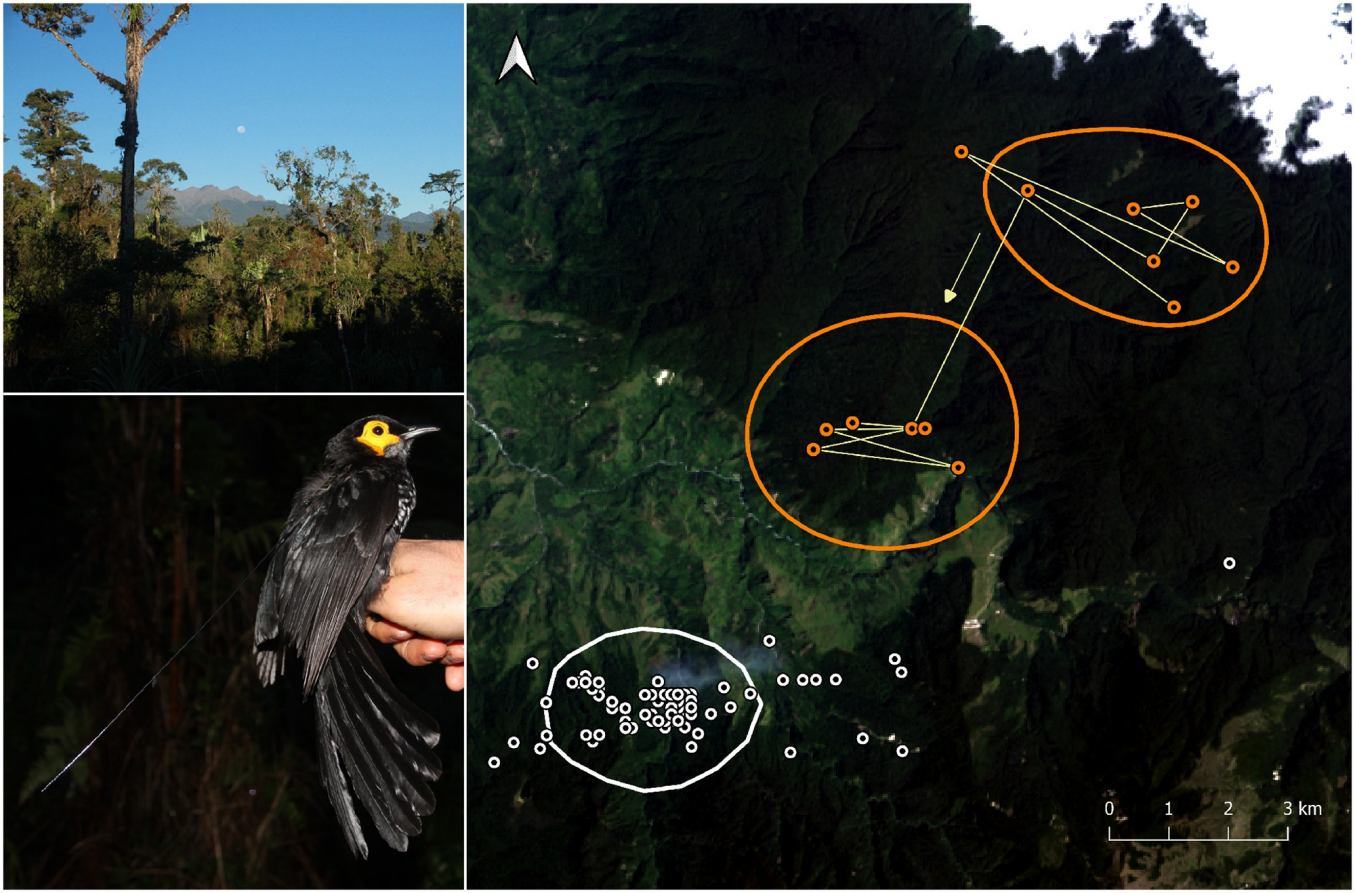
A home range shift by a Spangled Honeyeater revealed by satellite tracking data. Points from a stationary control transmitter (white) provide context for interpreting movement patterns of the honeyeater (orange). Side panels show montane forest habitat in New Guinea and a Spangled Honeyeater fitted with a transmitter. Photos: Knud Andreas Jønsson. Landsat-8 image courtesy of the U.S. Geological Survey.

Birds were caught using ornithological mist nets over the course of three field trips between September 2016 and December 2018. We set nets at active bowers of MacGregor’s Bowerbird (n = 2) and Archbold’s Bowerbird (n = 2). Birds were weighed upon capture. To ensure that the birds were as fit as possible upon release, they were given 0.5–1 h rest and provided with water in covered pet transport cages before further handling. Each bird was fitted with a 5 g solar-powered Platform Transmitter Terminal (Microwave Telemetry PTT-100). The mass of the transmitters corresponded to 2.7%–4.5% (mean = 3.9%) of the birds’ body mass. Transmitters were fitted to the birds like a backpack across the upper mantle, using a harness made from 2 mm braided nylon string (Fig 2). We lightly trimmed some mantle feathers to reduce obstruction of the solar cells. In 2017 and 2018, we took blood samples (for sexing) from the basilic vein in the wing from all individuals except for two male-plumaged bowerbirds. We did not take blood samples in 2016. Handling lasted approximately 45 min to 1 h per bird; birds were given 1 h rest in covered pet transport cages prior to release.

The transmitters were programmed to follow a 10 hours on-duty / 48 hours off-duty cycle. We enabled the XT^™^ feature, which allows transmission during the scheduled “off-duty” time if the transmitter is sufficiently charged. The geographic positions of the transmitters were obtained from ARGOS/CLS Service Argos30 [33]. Data are in S1 Dataset.

Interpreting satellite transmission data from sedentary birds poses the challenge of distinguishing actual movements from imprecision in the returned positions. To address this, we set up two stationary control transmitters, of the same model and with the same settings as the ones attached to the birds. The first was installed at a mountain site in the YUS Conservation Area (Table 1), where we attached it to the roof of a hut in a forest clearing at 1,700 masl. Steep mountain cliffs surrounded the clearing on two sides. It was activated from 14 November 2018 to 15 April 2019. The second control transmitter was installed in the coastal village of Nagada (Table 1), in central Madang Province. It was placed on the roof of a shed in a garden bordering the sea, where it was partly shaded by a large *Ficus* tree. It was activated from 3 December 2018 to 27 June 2019.

### Sexing tracked birds

Males and females of a given bird species may show different movement patterns arising from different breeding demands. Adult males of the bowerbirds are easily recognizable based on plumage characters. However, sexual dimorphism is much more subtle in the tracked honeyeater species, and immature male bowerbirds resemble adult females. We therefore genetically sexed birds for which blood samples were available (S1 Table in S1 Appendix). We used the primer pair 2550 F and 2718 R [34]. PCRs were run under 94°C for 3 min, followed by 30 cycles of 94°C for 45 sec; 55°C for 45 sec; 72°C for 55 sec; and, finally 72°C for 5 min. PCR products were subsequently visualized on an agarose gel (2%) and the heterogametic females (two fragments) and the homogametic males (one fragment) were identified.

### Analysis of tracking data

To test whether the movements of non-dispersing individual birds were detectable, we estimated and compared home ranges of tracked birds and stationary control transmitters. For this we used a high-quality subset of the obtained position data. Argos applies a seven-category quality ranking to each position; we analyzed only positions ranked among the three highest quality classes (“1,” “2,” and “3”). Class 1, 2, and 3 positions are reported to have respective estimated errors of 500–1500 m, 250–500 m, and < 250 m [33]. We analyzed only the ten individuals with ≥10 positions meeting this quality criterion [35]. We discarded all points over 50 km distant from the first obtained Argos location of Class 1, 2, or 3. For one individual, MacGregor’s Bowerbird 36508, the first acquired position was rated as Class 1 but was clearly erroneous (3,000 km from the release point), so the second acquired position was used instead. We used utilization distributions, as estimated with the bivariate normal kernel [36, 37], to define home ranges. This was implemented in the package adehabitatHR v0.4.18 [38] in R v3.5.2 [39]. We used the packages sp v1.4–5 [40, 41], fossil v0.4.0 [42], and rgdal v1.4–8 [43] to format the spatial data We then used the adehabitatHR functions ‘kernelUD’ (with smoothing and grid parameters/extent settings *h* = "href", *grid* = 100, *extent* = 1) and ‘getverticeshr’ to estimate home ranges of birds and controls as 95%, 90%, 80%, 70%, 60%, and 50% KUDs (kernel utilization distributions).

We also tested for seasonal variation in transmission data quality. Like many tropical regions, Papua New Guinea’s annual climatic regime can be roughly divided into a wet season and a dry season. To investigate whether rainfall (and by inference, cloud cover) affected the quantity and quality of transmission data, we used data from the CPC Merged Analysis of Precipitation (CMAP) [44]. CMAP merges data from several independent sources to estimate global monthly precipitation across a 2.5° latitude x 2.5° longitude global grid. We plotted these precipitation data and visually evaluated them against the transmission histories of all individuals with at least ten high-quality positions (n = 10), plus controls (n = 2).

### Permits and ethics statement

The fieldwork in Papua New Guinea was carried out under research permit numbers 99902341114 (A.H.R.), 99902341112 (K.H.B.), 99902260246 (T.E.O.), and 99902260244 (K.A.J.). Applications were approved by the PNG National Research Institute. Field sites were located on privately owned land, and access was arranged through the landowners. Samples were exported from Papua New Guinea under export permits issued by the Department of Environment and Conservation (permits 016210 [in 2016], 018208 [in 2017], and 019067 and 019069 [in 2018]). No protected species were sampled. Capture and sampling methods were approved by the Copenhagen Bird Ringing Center under the authority of the Danish Nature Agency (J.no. SN 302–009), and by the Institutional Ethics Committee of the New Guinea Binatang Research Centre.

## Results

### Tracking data

**Fig 3:**
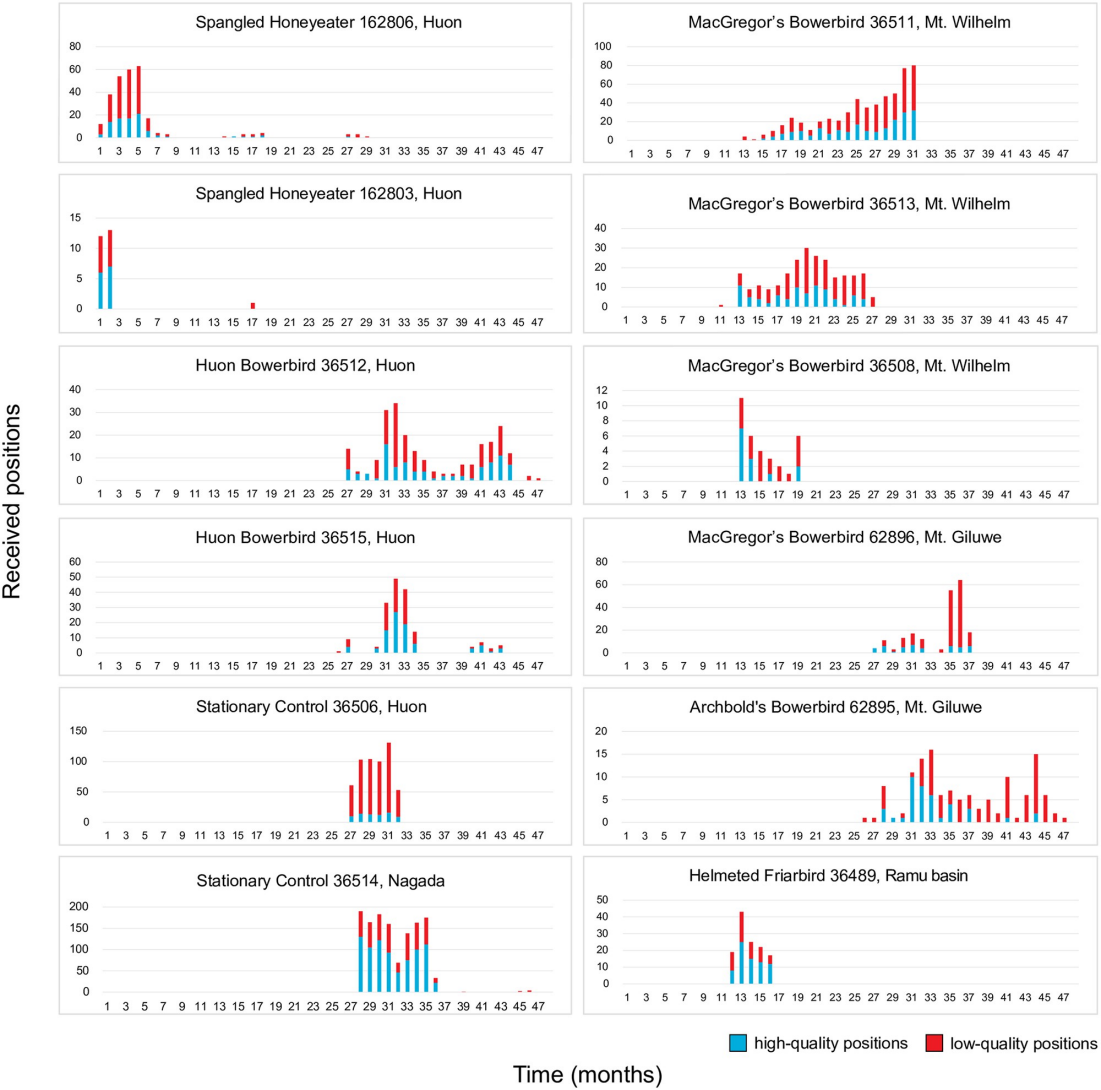
Transmission histories for tracked birds and stationary control transmitters. Time (in months) is measured on the x-axis; “1” corresponds to September 2016, and “47” corresponds to August 2020. All birds began transmitting within ten days after release. The y-axis measures number of positions obtained; note that scale varies between plots. Blue bars indicate high-quality points (Argos classes 1, 2, and 3); red bars indicate low-quality points (classes 0, A, B and Z). Three birds with ˂ 10 high-quality positions are excluded.

We obtained tracking data from all 13 birds that were fitted with satellite transmitters. Geographic positions obtained for individual birds are mapped in S1–S10 Figs in S1 Appendix, and transmission histories are plotted in Fig 3. For this study, we established a cutoff date for data collection at 13 October 2020; all individuals appear to have stopped transmitting by that date, except one Huon Bowerbird (36512). We obtained between one and 210 high quality locations (classes 1, 2, or 3) for each bird (mean = 57). Transmission periods—the length of time between the first and last transmissions—varied from four days to 29 months. No transmitter took more than ten days to send its first location fix after deployment. The two stationary control transmitters (S11, S12 Figs in S1 Appendix, Fig 3) respectively generated 74 and 810 high quality positions prior to their removal.

The number of obtained positions for individual birds generally decreased or remained roughly constant over the period from the first to the last transmission (Fig 3). MacGregor’s Bowerbird 36511 was the only individual in which the number of obtained positions steadily increased over time. There was little obvious change in position quality over time (Fig 3). Some individuals had complete, or near-complete transmission lapses lasting up to eight months (e.g. Spangled Honeyeater 162806, Huon Bowerbird 36515), before resuming transmission at a decreased rate.

We detected one compelling example of a dispersal, in Spangled Honeyeater 162803 in 2016 (Fig 2). This individual was released 11 September, and it transmitted seven high-quality positions from the same area (50% KUD 13 km2) until 1 October. From 3–17 October, it transmitted six high-quality positions from a new area approximately 4 km southwest of the release point. We received no further high-quality transmissions from it thereafter.

Out of thirteen birds tracked, three generated a substantial number of high-quality postions beyond the first year of tracking. Plotting combined tracking data from multiple years (S1, S5, S6 Figs in S1 Appendix) potentially obscures visual patterns of seasonal range shifts. Therefore, for MacGregor’s Bowerbird 36511 and Huon Bowerbird 36512, we separately plotted tracking data from the first and second years of activity (S13, S14 Figs in S1 Appendix). Huon Bowerbird 36515 did not generate enough positions during its second year of activity to warrant plotting, but we excluded the second-year positions to more clearly visualize data from the first year (S15 Fig in S1 Appendix). However, this exercise did not reveal any evidence of seasonal range shifts.

### Home range size estimates

There was little clustering of home range sizes among individuals from the same species or locality (S16, S17 Figs in S1 Appendix). Individuals from Mt. Wilhelm tended to have smaller home ranges than those from Huon, but the difference between the 95% KUD home ranges was not significant (Welch’s *t*-test, *t*(3.37) = -1.7, *p* = .173). The lowland control transmitter at Nagada had a 95% home range of 5.5 km^2^, and a 50% home range of 0.2 km^2^; these were the lowest estimates in the study. The montane control in the YUS Conservation Area had a 95% home range of 50 km^2^, and a 50% home range of 7 km^2^, estimates larger than those for many of the birds. There was no difference in the mean 95% KUD home ranges of birds and control transmitters (Welch’s *t*-test, *t*(1.15) = 0.6, *p* = .669). The dispersing Spangled Honeyeater 162803 was not considered in these comparisons. We repeated the tests with a more stringent data threshold, excluding KUD estimates from all individuals with fewer than 30 high-quality positions as recommended by Seaman et al. [45]. This resulted in the exclusion of one additional individual, but did not change the basic results.

### Seasonal variation in transmission data quality

Monthly precipitation is plotted against summarized monthly transmission data in S18A, S18B Figs in S1 Appendix. Overall, transmission frequency does not decline with increased rainfall, and there is a slight trend of increased transmission frequency during the wet season for Huon birds. There is no clear relationship between position quality and rainfall.

## Discussion

### Detecting movements

**Fig 4:**
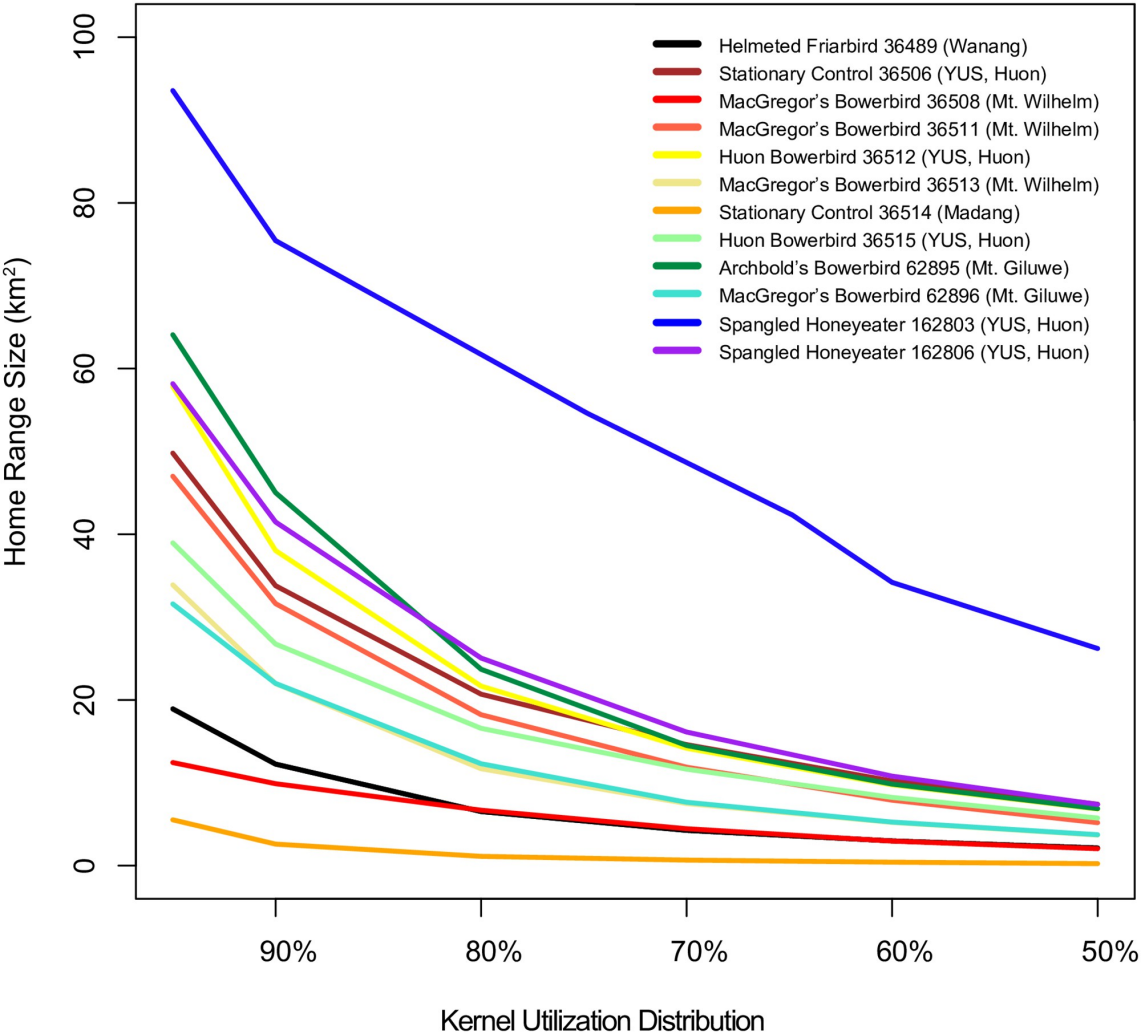
Home range size estimates for tracked birds and stationary control transmitters. 95%–50% KUDs (kernel utilization distributions) are plotted. The large estimate for Spangled Honeyeater 162803 derives from the effective summing of two different home ranges, the result of a dispersal event (Fig 2). Most other individuals have home ranges estimates falling between those of the stationary control transmitters in the lowlands (36514) and highlands (36506), indicating that birds are highly sedentary.

We detected just one instance of a dispersal among the tracked individuals: a sudden 4 km range shift by a Spangled Honeyeater in the mountains of the Huon Peninsula (Fig 2). This individual generated only 13 high-quality points in total, but the spatiotemporal clustering of the points strongly suggests that this was an actual dispersal rather than an artifact of data error. All points, as well as the inferred dispersal route, were inside mature montane forest at 2,100–3,100 masl, well within its known elevational range [29]. There were no clear dispersals observed for any of the other birds in the study. Most individuals have high-quality positions indicating long-distance movements of 5–10 km or more (S1–S12 Figs in S1 Appendix), but we interpret this as data error. If birds were actually ranging widely, we would expect to see that the outlying points were clustered (or ‘doubled-up’) to indicate that a bird had remained in the same area in between transmissions. We do not see this, and the outlying points are much more readily interpretable as random and artificial scatter around a real core home range. The stationary control transmitters produced similar sets of outlying positions with high quality ratings (S11, S12 Figs in S1 Appendix). Most bird home range sizes fell somewhere in between estimates for the two stationary controls (Fig 4). On the Huon Peninsula, where we both tracked birds and set up a control, bird and control home ranges were very similar, apart from the dispersing Spangled Honeyeater (S17 Fig in S1 Appendix).

Seasonal movement has previously been observed in two of the species in our study, MacGregor’s Bowerbird [30] and Helmeted Friarbird [32], and it is possible for the other tracked species, as their movements are not well studied. About half the tracked birds produced long positional data series that extended across seasonal transitions (S18 Fig in S1 Appendix). We were able to evaluate whether these individuals (including three MacGregor’s Bowerbirds) made movements corresponding with transitions between wet and dry seasons. The dispersal by a single Spangled Honeyeater conceivably represents a ‘seasonal movement,’ as it was made during the transition from the dry to wet seasons. However, the data series was not long enough to detect any additional seasonal pattern, and the other Spangled Honeyeater tracked in the same area made no similar movements (S9 Fig in S1 Appendix). No other individuals of any species established new home ranges between seasons, and we could not detect any seasonal shifts in the distribution of points within individual home ranges. Overall, apart from a single exception, the birds in our study were highly sedentary.

Satellite tracking is a powerful tool for following the global movements of long-distance migrants [e.g., 8, 9], but detecting the fine-scale movement patterns of resident passerines demands much more precise and accurate positional data. We show that relatively small movements of a few kilometers will often be detectable, meaning that there is excellent potential for measuring individual dispersals and seasonal movements in small tropical forest species. However, we found it impossible to trace individual birds’ movements through their home ranges, due to the small size of those home ranges and the margin of error in the transmitted positions. Our highland and lowland stationary control transmitters contrasted strongly in number of positions returned and estimated home range, suggesting that performance varies significantly with locality. In this case, the disparity may result from the transmitters’ respective locations in flat coastal versus rugged mountain terrain. Topographical complexity is known to affect transmitter performance in GPS telemetry [46, 47], although this may be less important in Argos telemetry [48]. The variable transmitter performance complicates comparison of home range size between individuals at different sites. Activating stationary transmitters at each study locality could help control for this variation, but for now this is a very expensive proposition.

### Performance over time

Determining the fine-scale movement patterns of tropical passerines depends upon obtaining transmission data at frequent intervals over long periods of time. If transmitters work for a long time, there are better chances for detecting regular seasonal movements and uncommon dispersal events; and more data points can improve home range estimates. Six of thirteen birds in our study transmitted high quality positions for over a year. Transmission frequency declined over time for most of these (though transmission quality remained roughly constant), and some had long periods with few or no transmissions (Fig 3). These periodic transmission lapses, most evident in birds from the Huon, appear to occur during the dry season (S18 Fig in S1 Appendix). We hypothesized that rainy, overcast conditions might inhibit transmissions, but several birds actually transmitted more frequently in the wet season. We can only speculate as to why that is. Possibly birds make use of brief clear spells during the wet season to sun themselves in the upper canopy, charging the solar transmitters and increasing transmission rates.

It is also unclear why some transmission series cut off abruptly, and why transmission frequency declined over time in some cases. In Huon birds, there does not seem to be a slow, steady decline over time; rather, transmitters seem to work through the first rainy season, but then transmit at lower frequency for the rest of their lifespans (S18 Fig in S1 Appendix). One explanation for this pattern is that birds are molting their trimmed mantle feathers, and the new feathers significantly obscure the solar chargers. The overall low longevity and variable performance of the transmitters in this system is a real obstacle, and it is difficult to pinpoint whether the challenges lie with the technology, the birds, or the environment. Long-term experimental tracking and monitoring of predictably stationary animals—birds in aviaries or around permanent field stations—may provide some answers.

### Current and future prospects

Our study shows that modern satellite tracking technology can measure short dispersals and seasonal movements by resident passerine birds, and therefore has considerable potential to elucidate the movement biology of small tropical forest animals. At the same time, our field efforts demonstrate some significant methodological limitations. Total transmission periods were often short, and in other cases transmission frequency declined over time. There were sometimes long lapses between transmissions; precision and accuracy of data were limited, and varied between localities; and it was difficult to capture high numbers of birds suitable for tracking. Some of these roadblocks will be diminished as transmitters continue to get smaller and cheaper. The five-gram tags we used will soon be replaced by a two-gram model, meaning that the minimum body mass for tracked birds will drop to around 40 g. This will greatly increase the number of species available for tracking efforts. Based upon a body mass index for all bird species [49, 50], there are 330 passerine species worldwide weighing over 100 g; at 40 g, this leaps to 1,477 species. Thus, while we have provided a proof of concept for satellite tracking tropical forest passerines, scaling up tracking of earth’s faunal diversity will imminently shift from being possible to being a practical and effective new path for movement biology.

## Supporting information

S1 Appendix Supporting figures and table. (PDF)

S1 Dataset Movement data. (TXT)
