# Supplementary material for: Satellite tracking resident songbirds in tropical forests
Source: PLoS One. 2022 Dec 30;17(12):e0278641. doi: 10.1371/journal.pone.0278641 (PMC9803307; doi:10.1371/journal.pone.0278641)
Supplement: S1 Appendix — (PDF) [file pone.0278641.s001.pdf]

Supporting Information for

Satellite tracking resident songbirds in tropical forests

Contents

S1–15 Figs. Obtained positions and home range polygons for tracked birds and stationary control transmitters. Small circles indicate high-quality positions (Argos classes 1, 2, and 3). Lines reflect the temporal sequence of the positions. Home range polygons are superimposed as light blue rings; thick, medium, and narrow rings represent 50%, 75%, and 95% kernel utilization distributions, respectively. Three birds with < 10 high-quality positions are excluded. Landsat-8 images courtesy of the U.S. Geological Survey.

S1 Fig. MacGregor's Bowerbird *Amblyornis macgregoriae***,** Transmitter 36511. Mt. Wilhelm.
S2 Fig. MacGregor's Bowerbird *Amblyornis macgregoriae***,** Transmitter 36508. Mt. Wilhelm.
S3 Fig. MacGregor's Bowerbird *Amblyornis macgregoriae***,** Transmitter 36513. Mt. Wilhelm.
S4 Fig. MacGregor's Bowerbird *Amblyornis macgregoriae***,** Transmitter 62896. Mt. Giluwe.
S5 Fig. Huon Bowerbird *Amblyornis germana***,** Transmitter 36512. YUS Conservation Area, Huon.
S6 Fig. Huon Bowerbird *Amblyornis germana***,** Transmitter 36515. YUS Conservation Area, Huon.
S7 Fig. Archbold's Bowerbird *Archboldia papuensis***,** Transmitter 62895. Mt. Giluwe.
S8 Fig. Spangled Honeyeater *Melipotes ater***,** Transmitter 162803. YUS Conservation Area, Huon.
S9 Fig. Spangled Honeyeater *Melipotes ater***,** Transmitter 162806. YUS Conservation Area, Huon.
S10 Fig. Helmeted Friarbird *Philemon buceroides***,** Transmitter 36489. Wanang, Ramu River basin.
S11 Fig. Stationary control**,** Transmitter 36506. YUS Conservation Area, Huon.
S12 Fig. Stationary control**,** Transmitter 36514. Madang.
S13A Fig. MacGregor's Bowerbird 36511 year 1 tracking data (11 Nov 2017 – 10 Nov 2018).
S13B Fig. MacGregor's Bowerbird 36511 year 2 tracking data (11 Nov 2018 – 10 Nov 2019).
S14A Fig. Huon Bowerbird 36512 year 1 tracking data (7 Nov 2018 – 6 Nov 2019).
S14B Fig. Huon Bowerbird 36512 year 2 tracking data (7 Nov 2019 – 6 Nov 2020).
S15 Fig. Huon Bowerbird 36515 year 1 tracking data (8 Nov 2018 – 7 Nov/2019).

S16 Fig. Home range size estimates as in Fig 4, color-coded by species.

S17 Fig. Home range size estimates as in Fig 4, color-coded by study area.

S18A–B Figs. Transmission histories for tracked birds and stationary control transmitters as in Fig 3, plotted against monthly precipitation (mm/day). Precipitation data is from CPC Merged Analysis of Precipitation (CMAP) (Xie & Arkin, 1997). Study areas span two cells of the CMAP geographic grid with different rainfall data, which is reflected in the plots (Mt. Giluwe birds versus all others).

S18A Fig. Birds from Huon; stationary control transmitters from Huon and Nagada.
S18B Fig. Birds from Mt. Wilhelm, Mt. Giluwe, and the Ramu basin.

S1 Table. Genetic sex information for tracked birds.

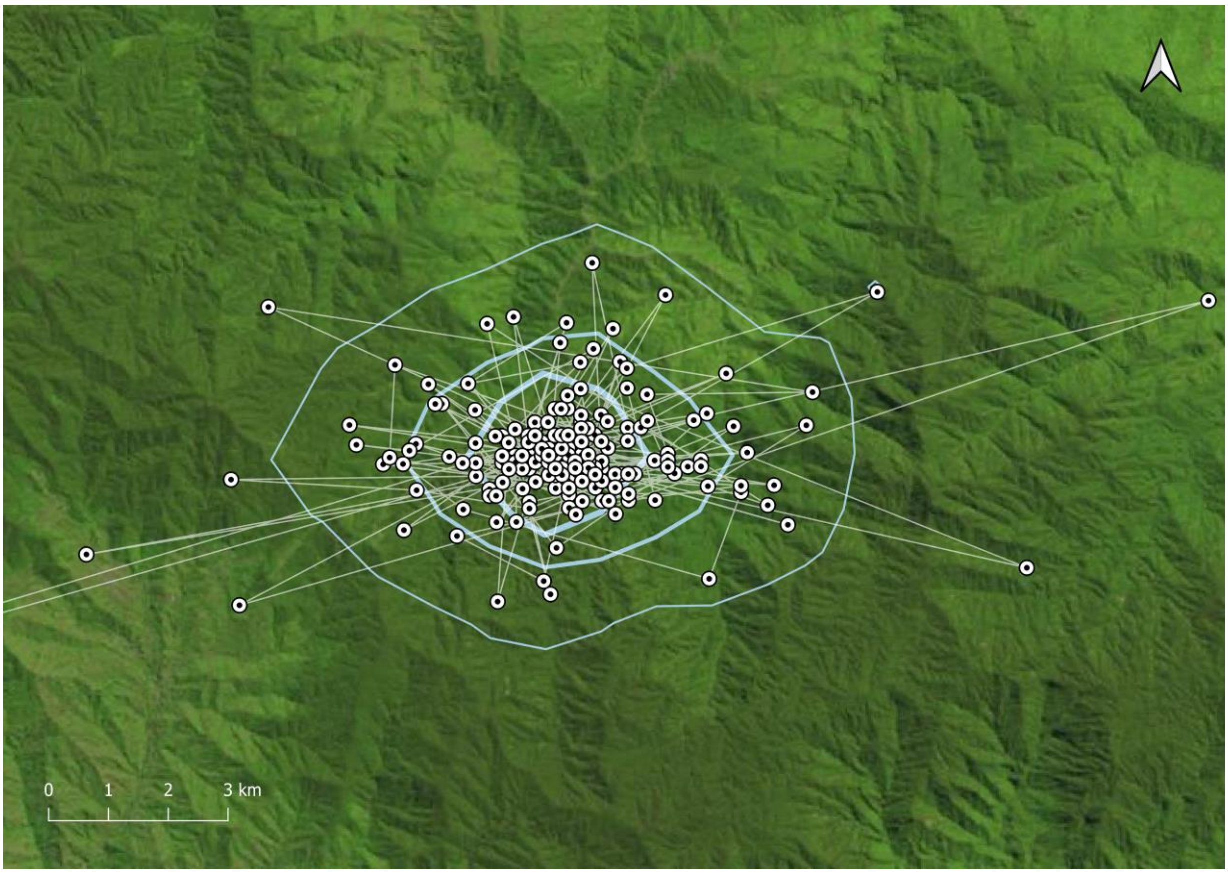

S1 Fig. MacGregor's Bowerbird *Amblyornis macgregoriae***,** Transmitter 36511. Mt. Wilhelm.

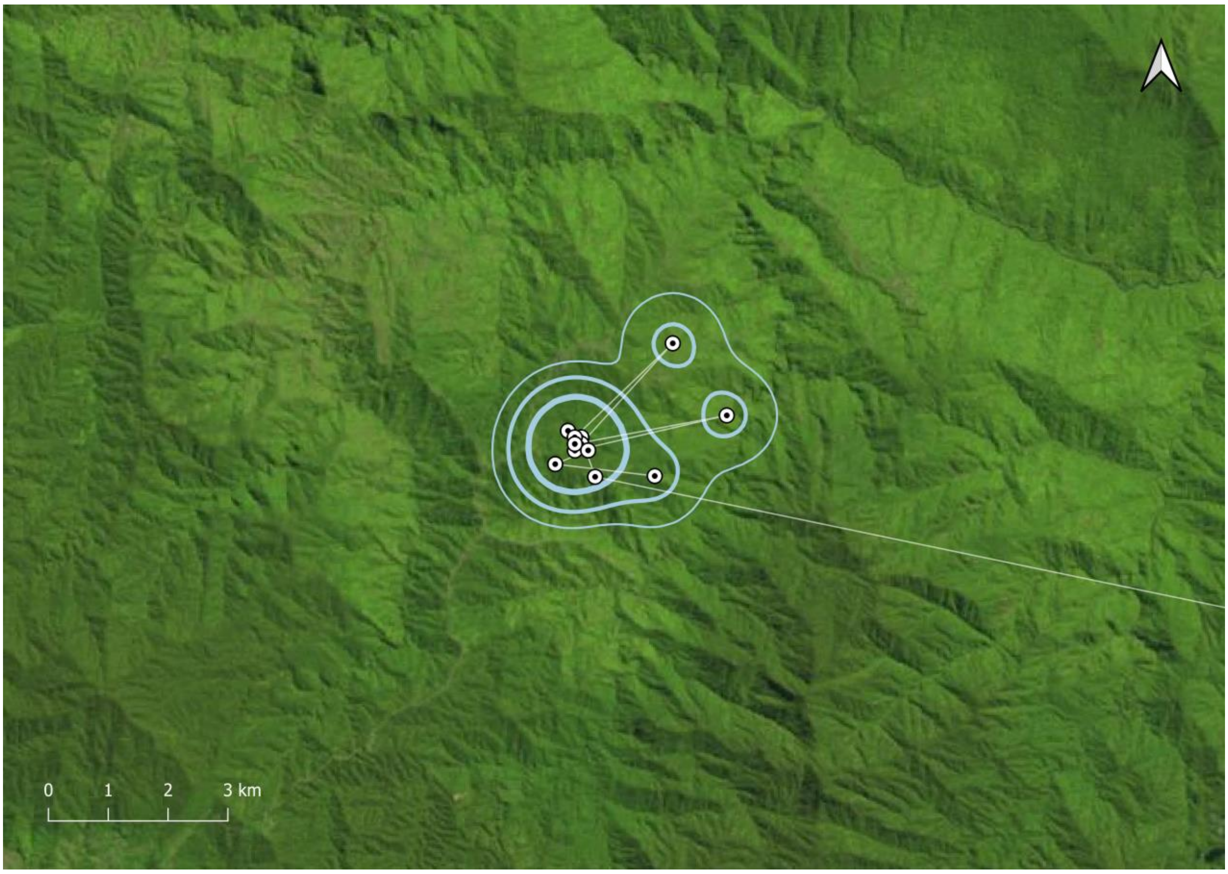

S2 Fig. MacGregor's Bowerbird *Amblyornis macgregoriae***,** Transmitter 36508. Mt. Wilhelm.

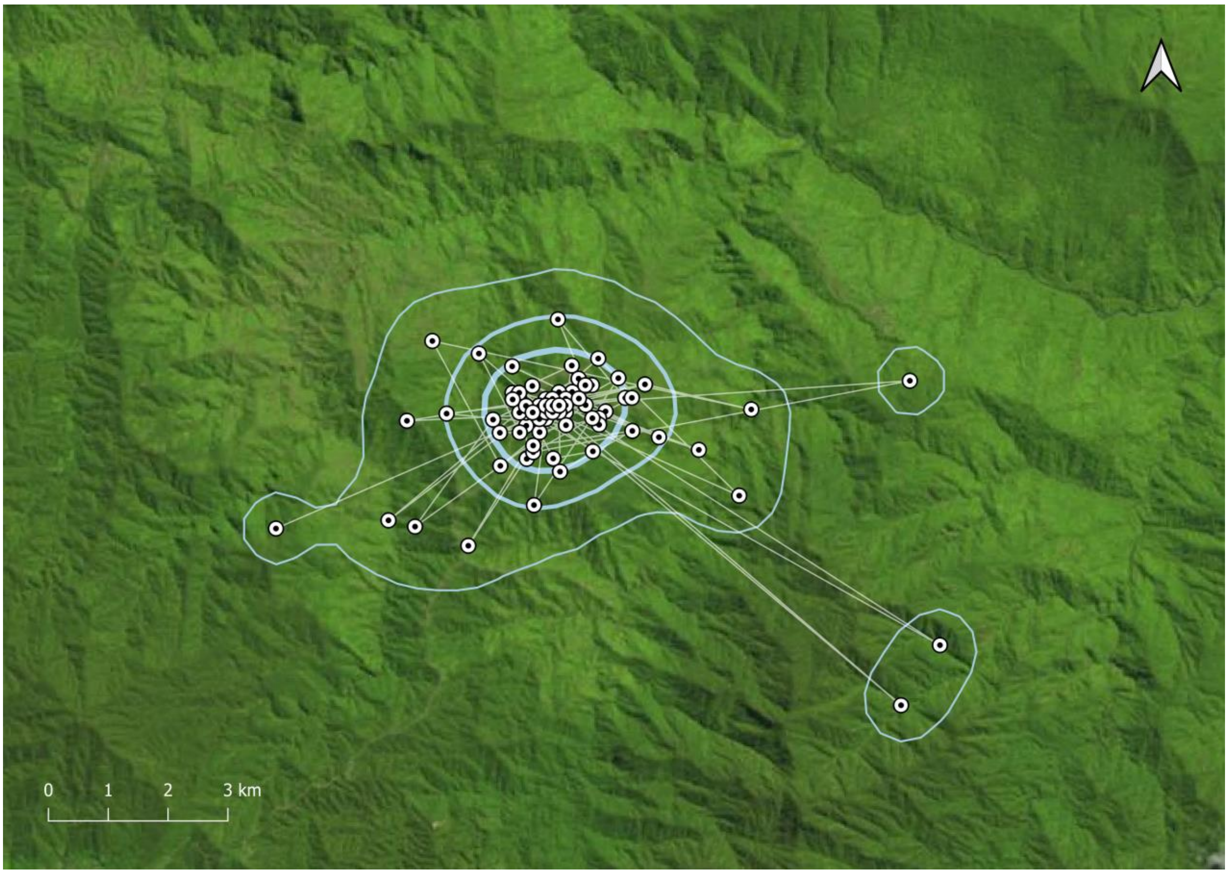

S3 Fig. MacGregor's Bowerbird *Amblyornis macgregoriae***,** Transmitter 36513. Mt. Wilhelm.

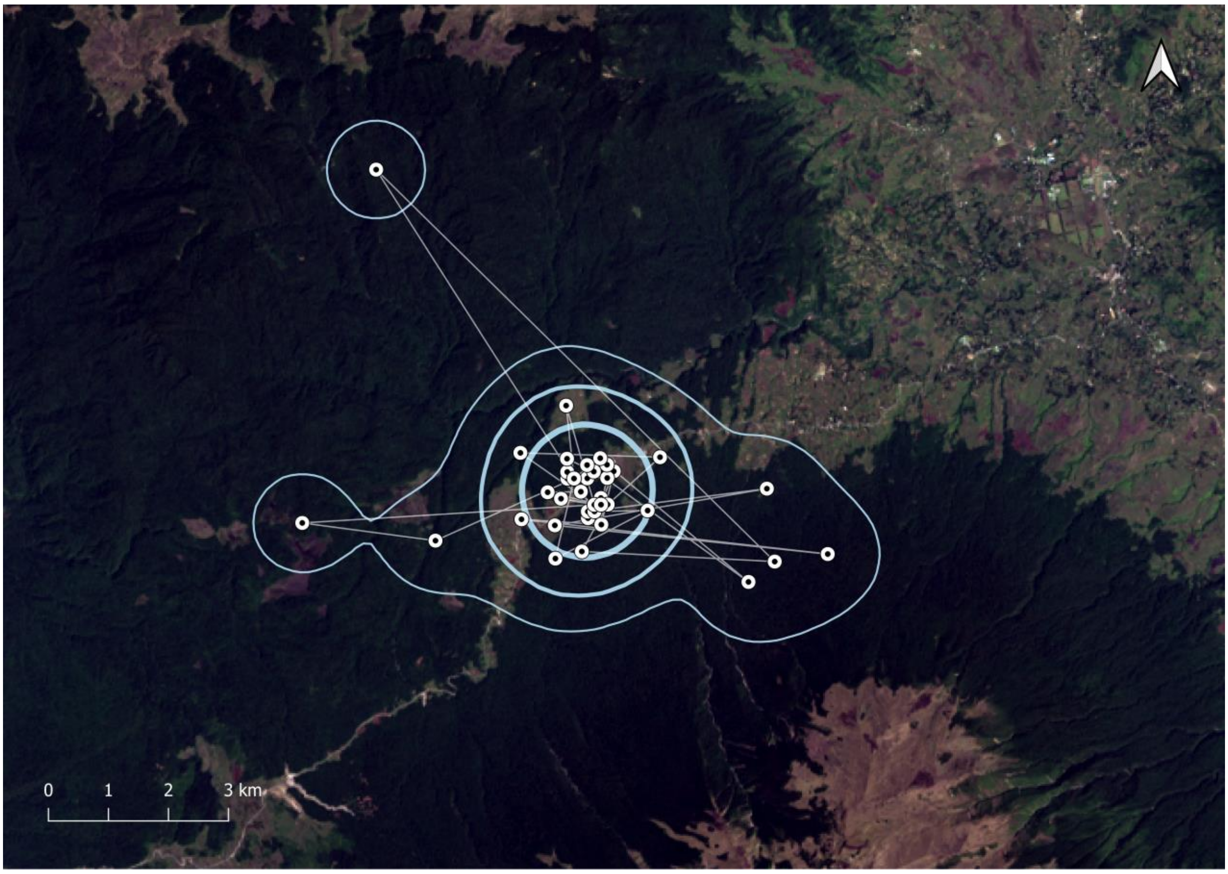

S4 Fig. MacGregor's Bowerbird *Amblyornis macgregoriae***,** Transmitter 62896. Mt. Giluwe.

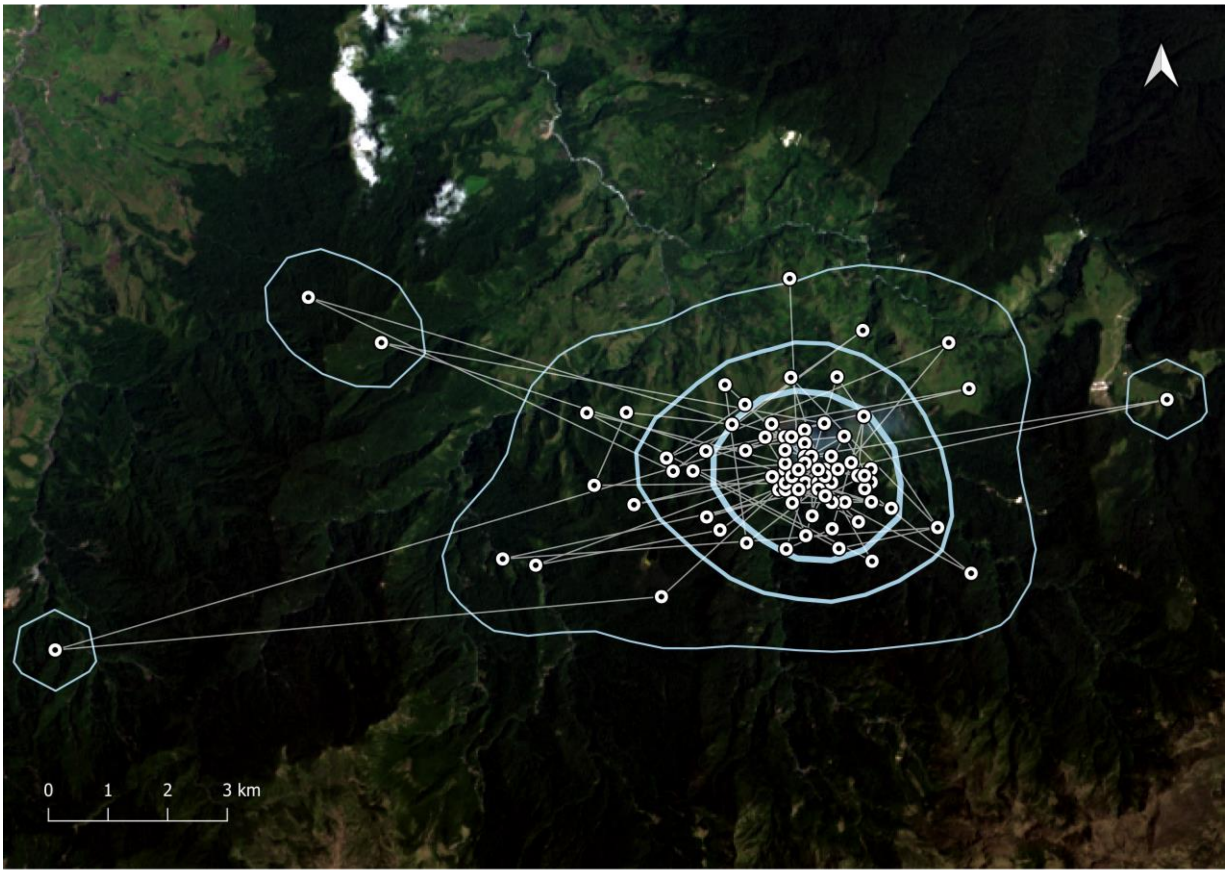

S5 Fig. Huon Bowerbird *Amblyornis germana***,** Transmitter 36512. YUS Conservation Area, Huon.

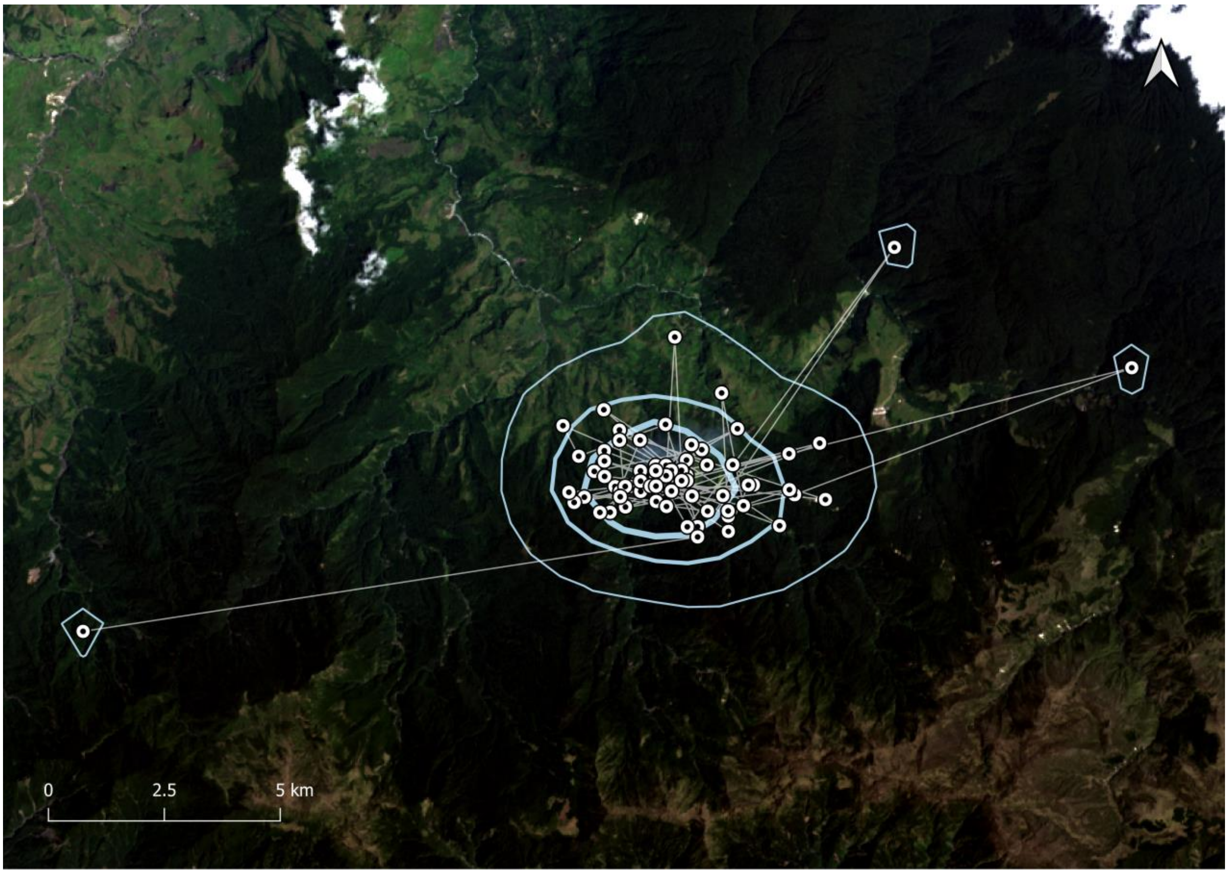

S6 Fig. Huon Bowerbird *Amblyornis germana***,** Transmitter 36515. YUS Conservation Area, Huon.

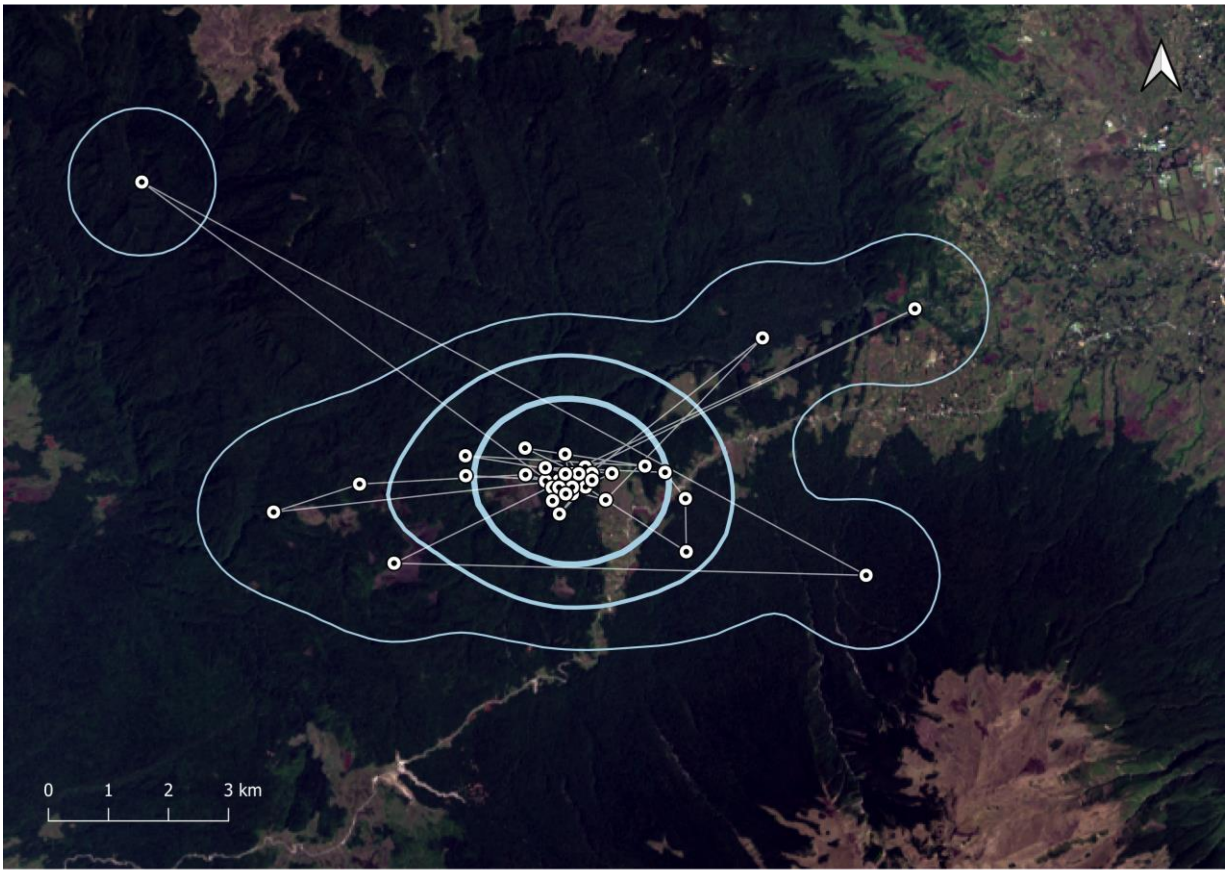

S7 Fig. Archbold's Bowerbird *Archboldia papuensis***,** Transmitter 62895. Mt. Giluwe.

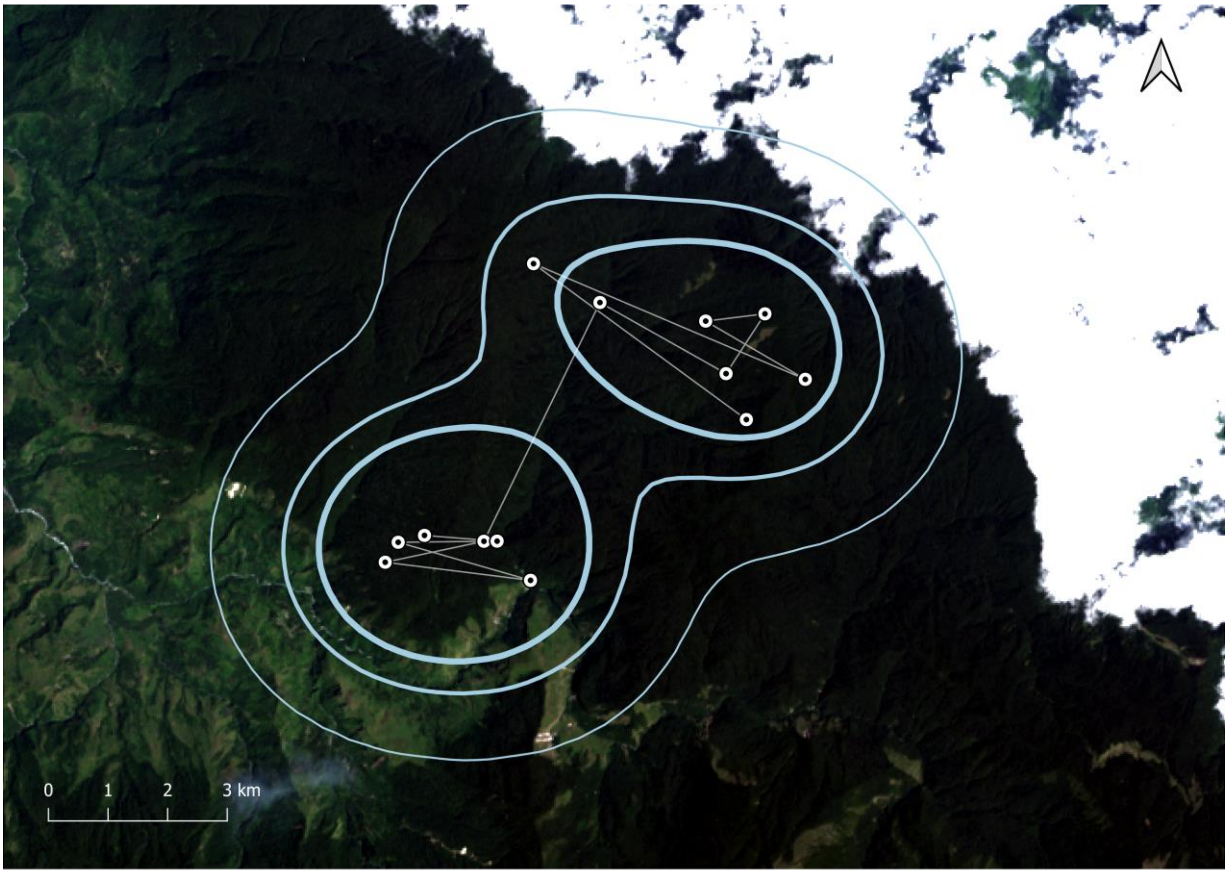

S8 Fig. Spangled Honeyeater *Melipotes ater***,** Transmitter 162803. YUS Conservation Area, Huon.

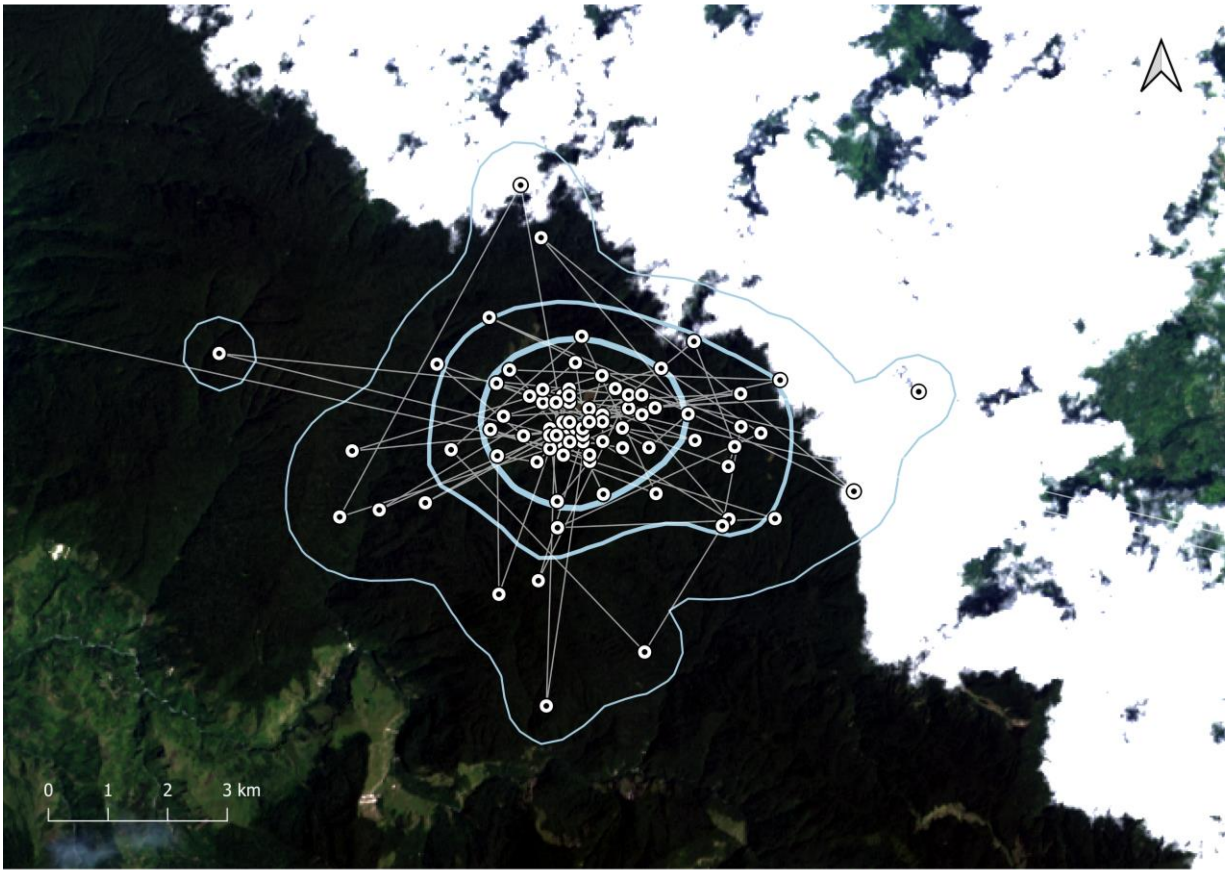

S9 Fig. Spangled Honeyeater *Melipotes ater***,** Transmitter 162806. YUS Conservation Area, Huon.

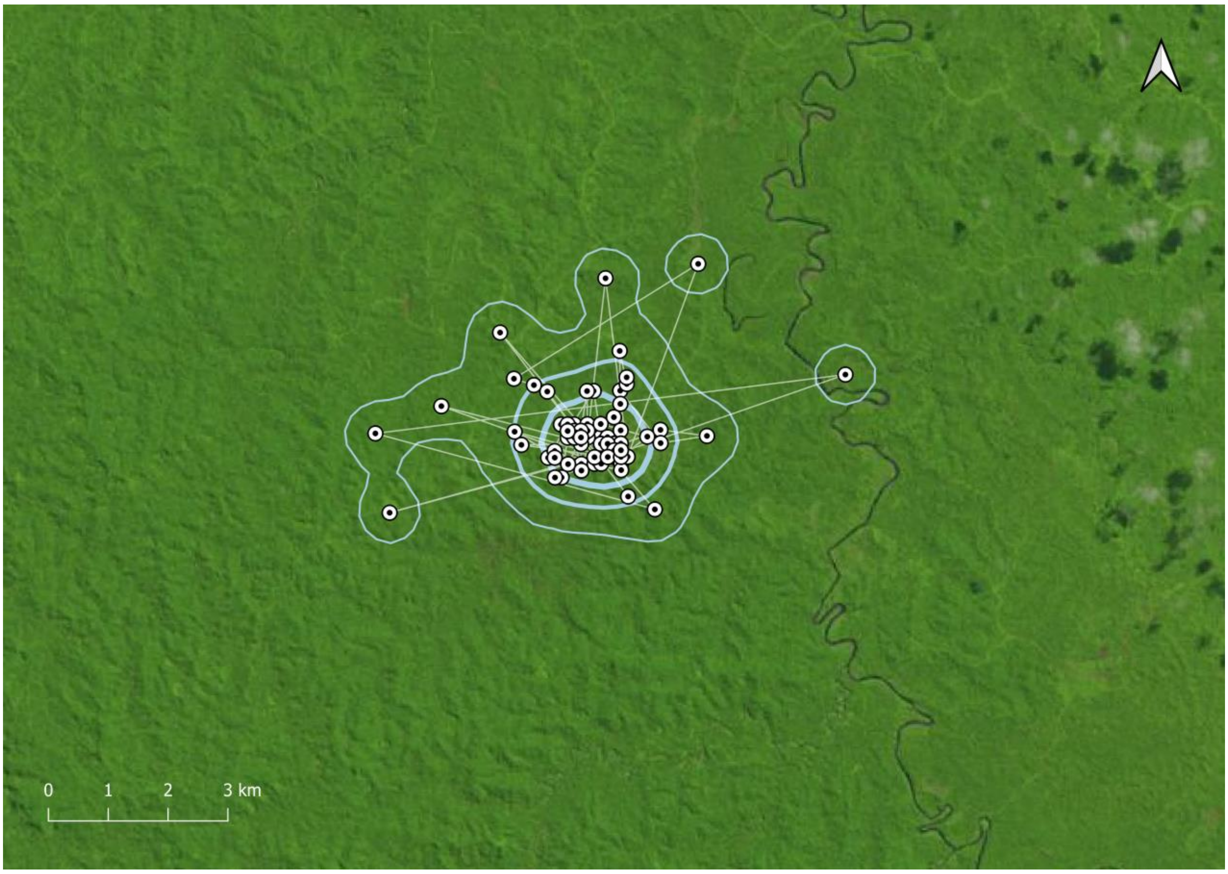

S10 Fig. Helmeted Friarbird *Philemon buceroides***,** Transmitter 36489. Wanang, Ramu River basin.

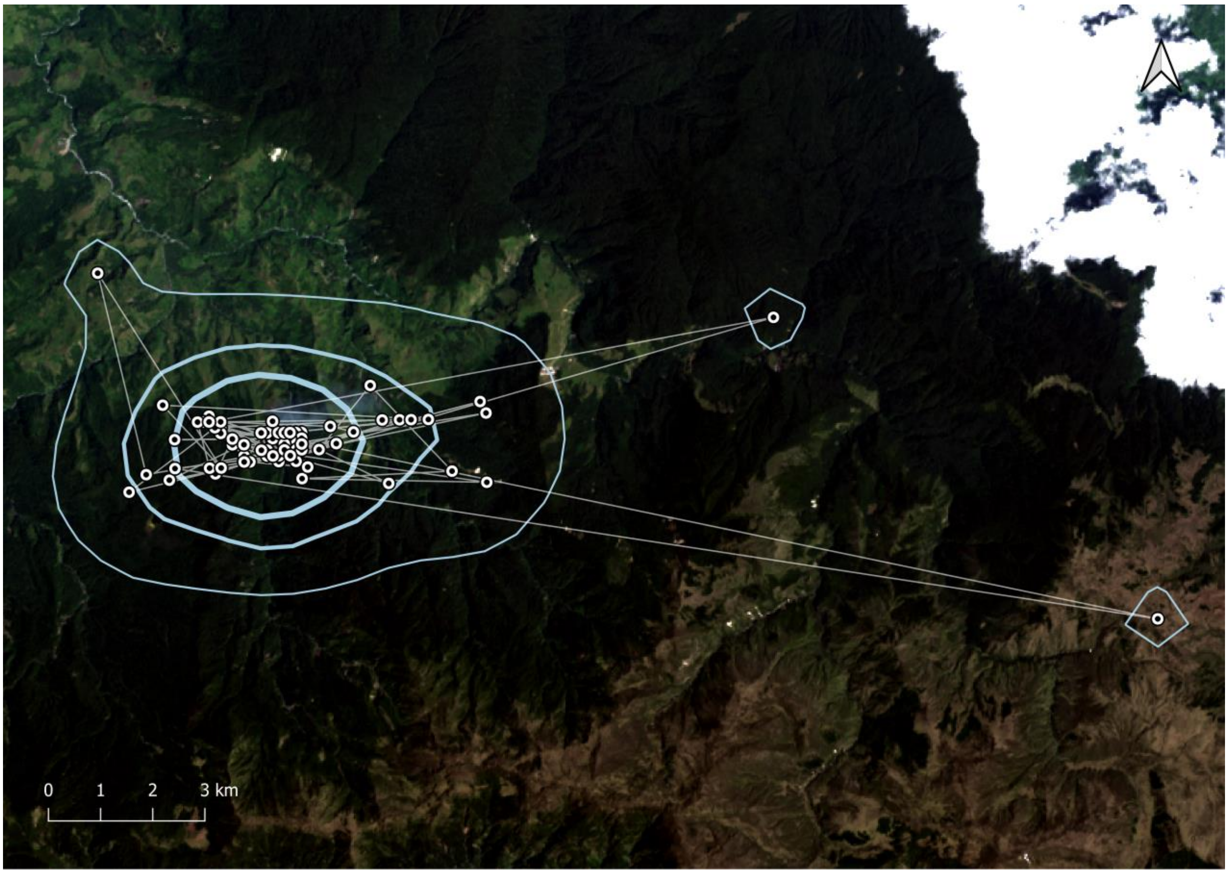

S11 Fig. Stationary control**,** Transmitter 36506. YUS Conservation Area, Huon.

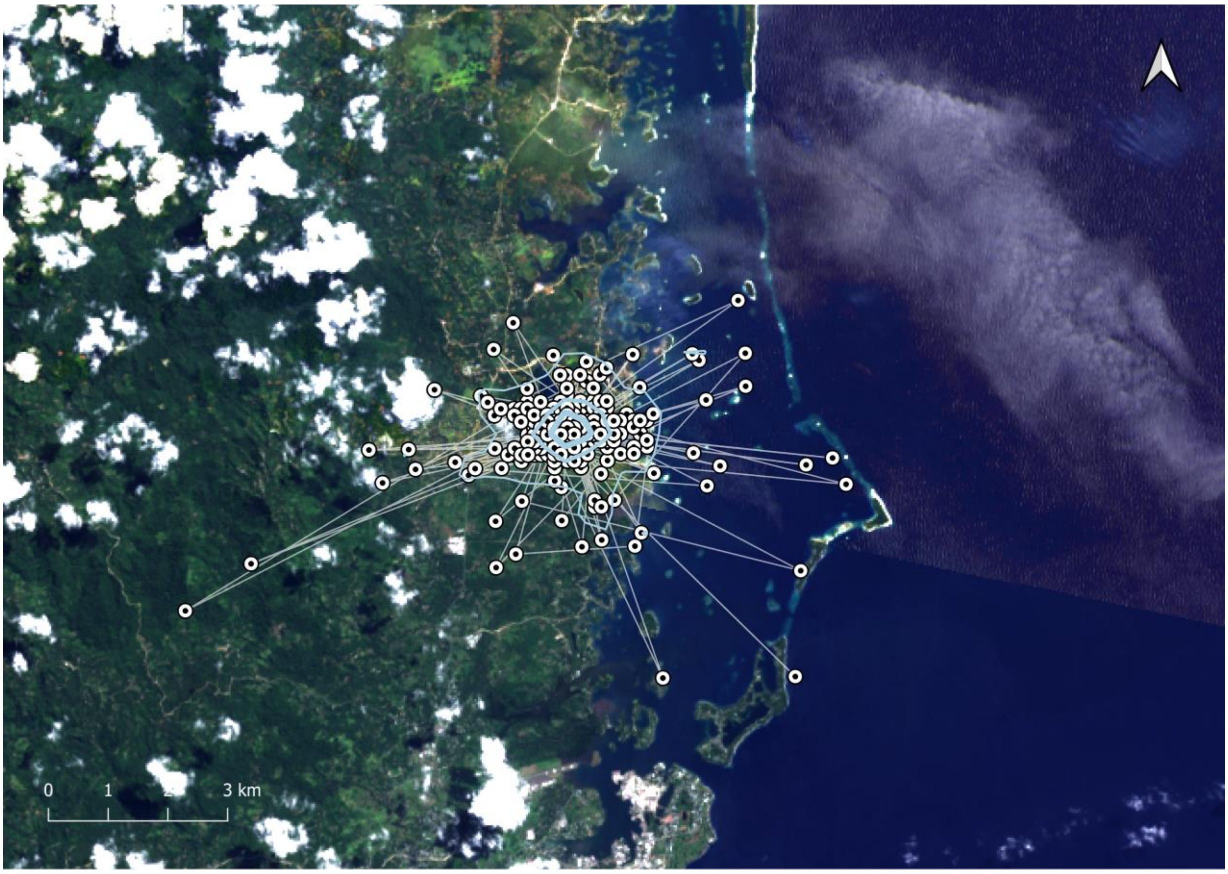

S12 Fig. Stationary control**,** Transmitter 36514. Madang.

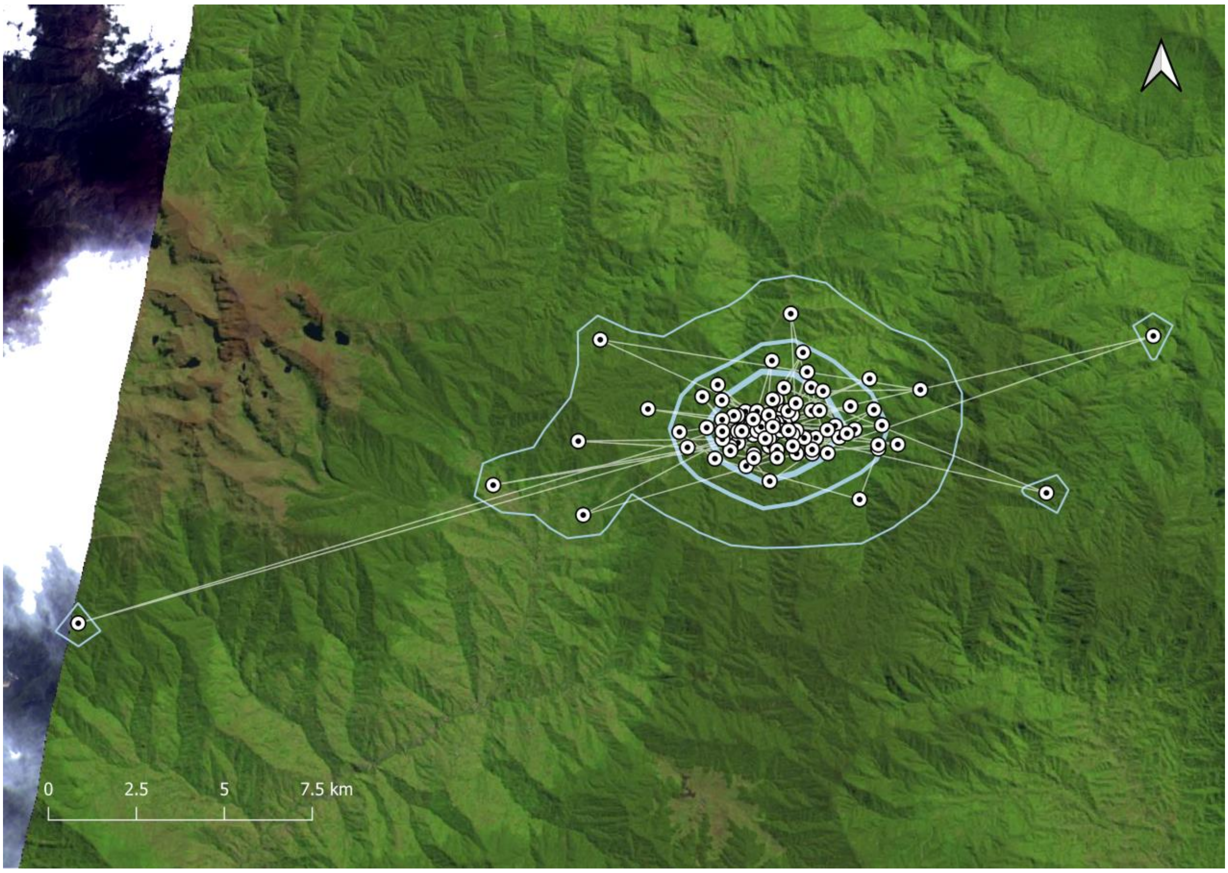

S13A Fig. MacGregor's Bowerbird 36511 year 1 tracking data (11 Nov 2017 – 10 Nov 2018).

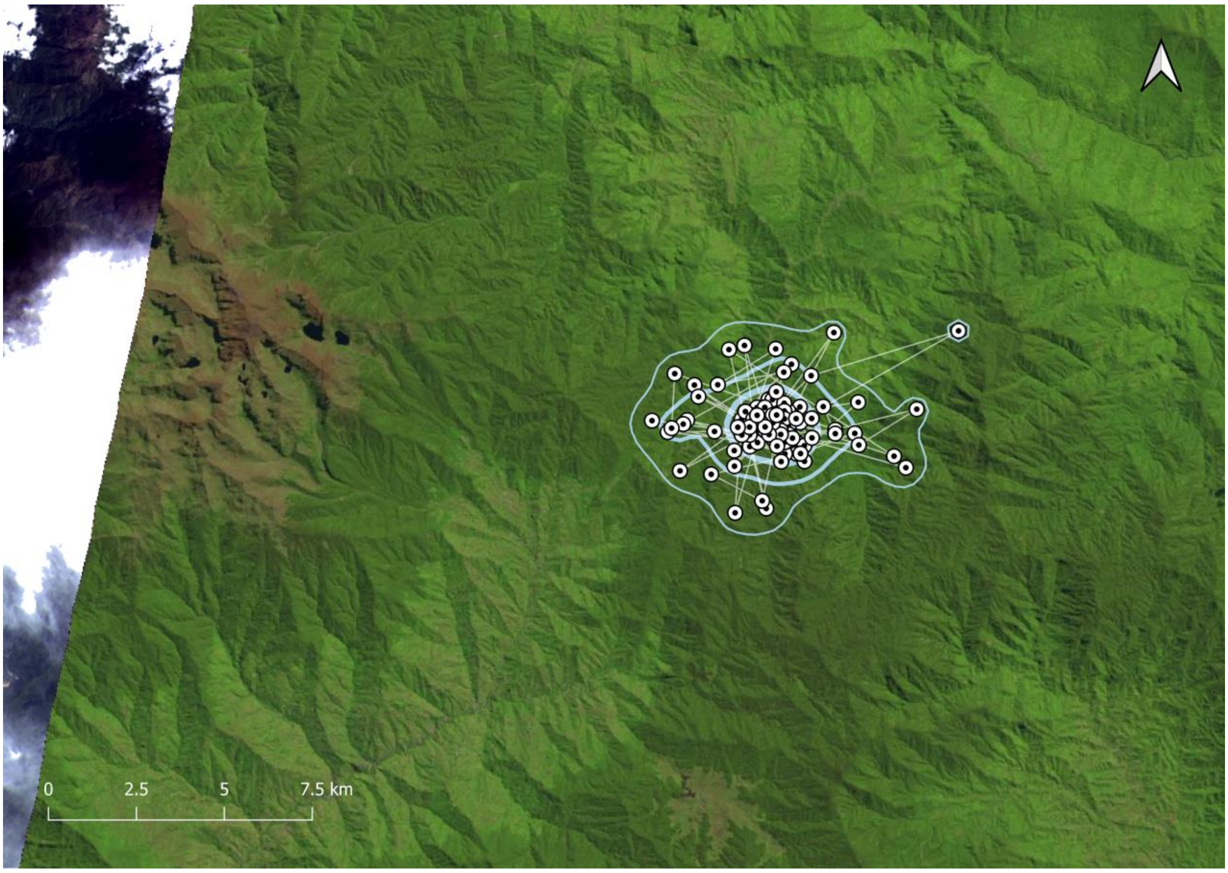

S13B Fig. MacGregor's Bowerbird 36511 year 2 tracking data (11 Nov 2018 – 10 Nov 2019).

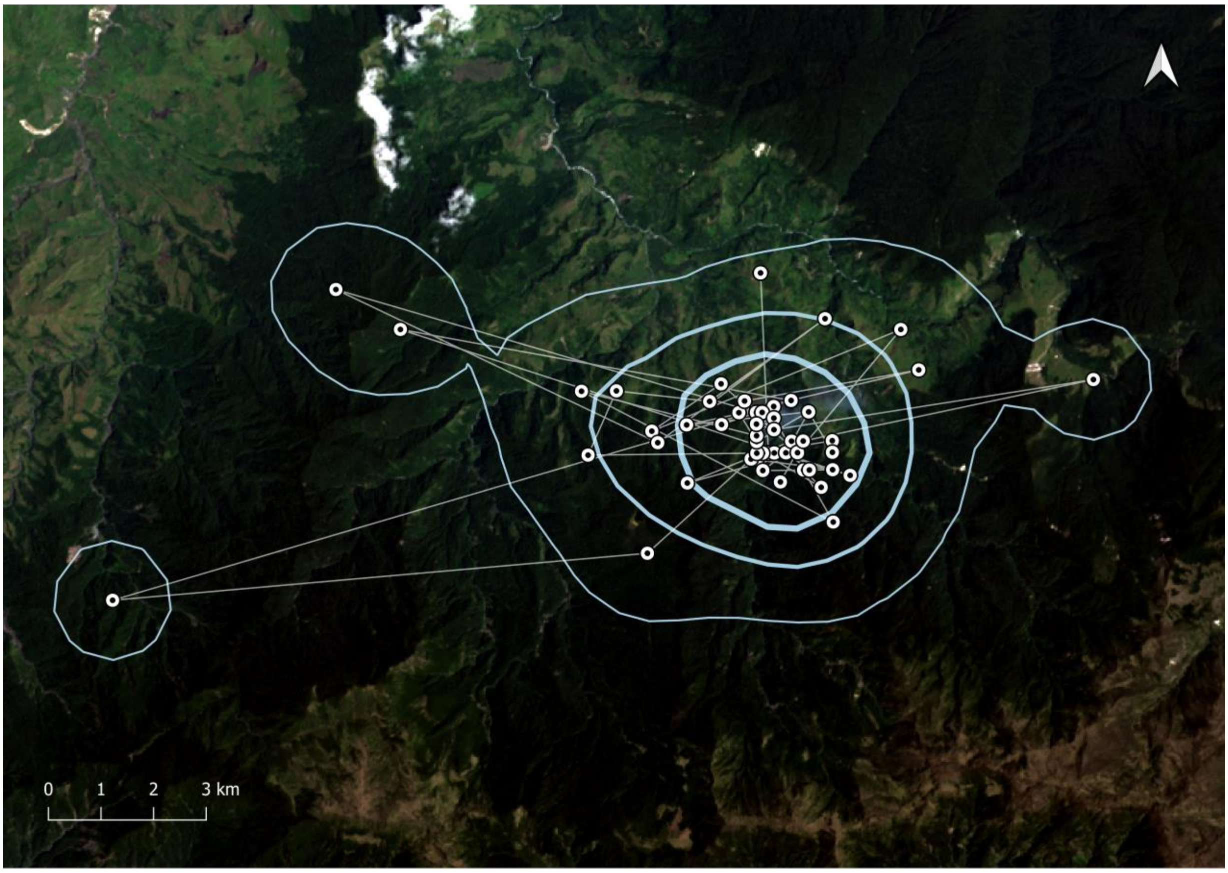

S14A Fig. Huon Bowerbird 36512 year 1 tracking data (7 Nov 2018 – 6 Nov 2019).

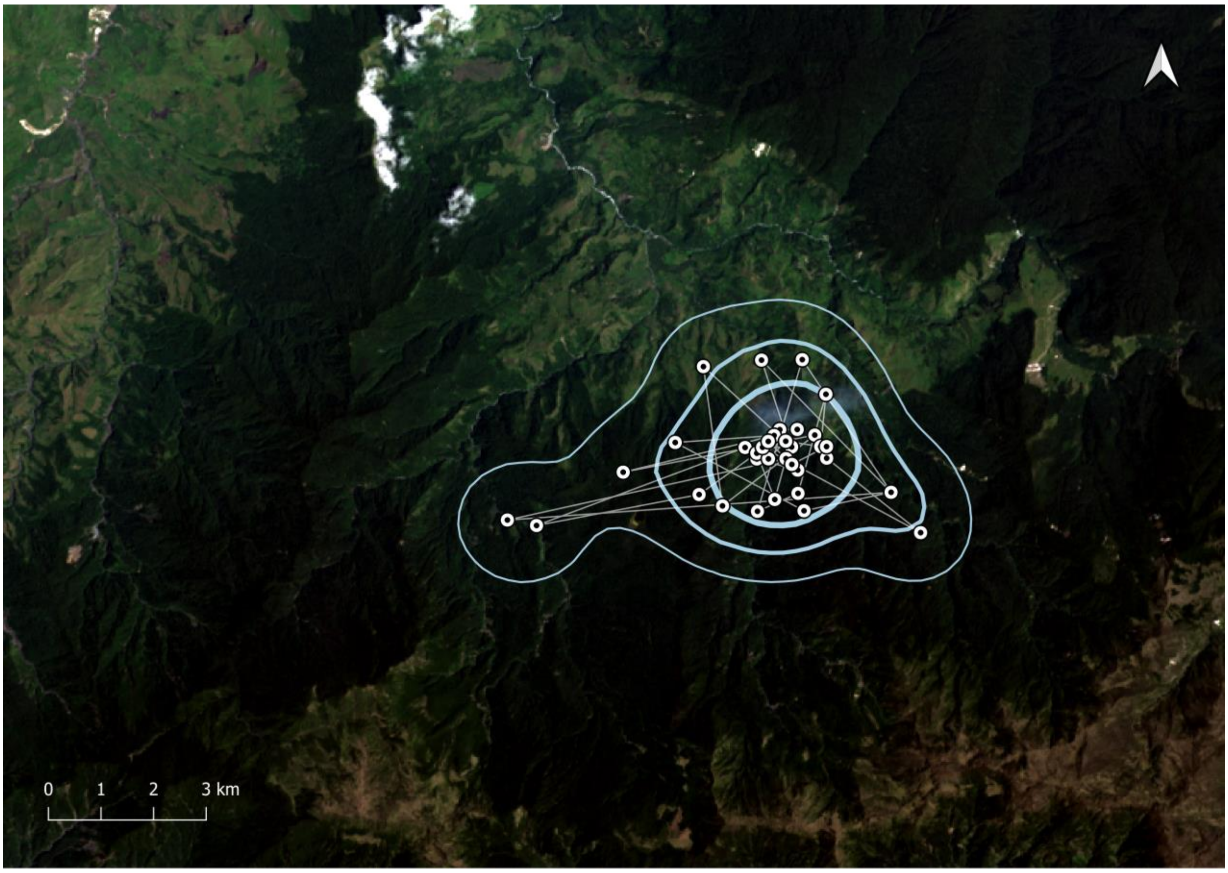

S14B Fig. Huon Bowerbird 36512 year 2 tracking data (7 Nov 2019 – 6 Nov 2020).

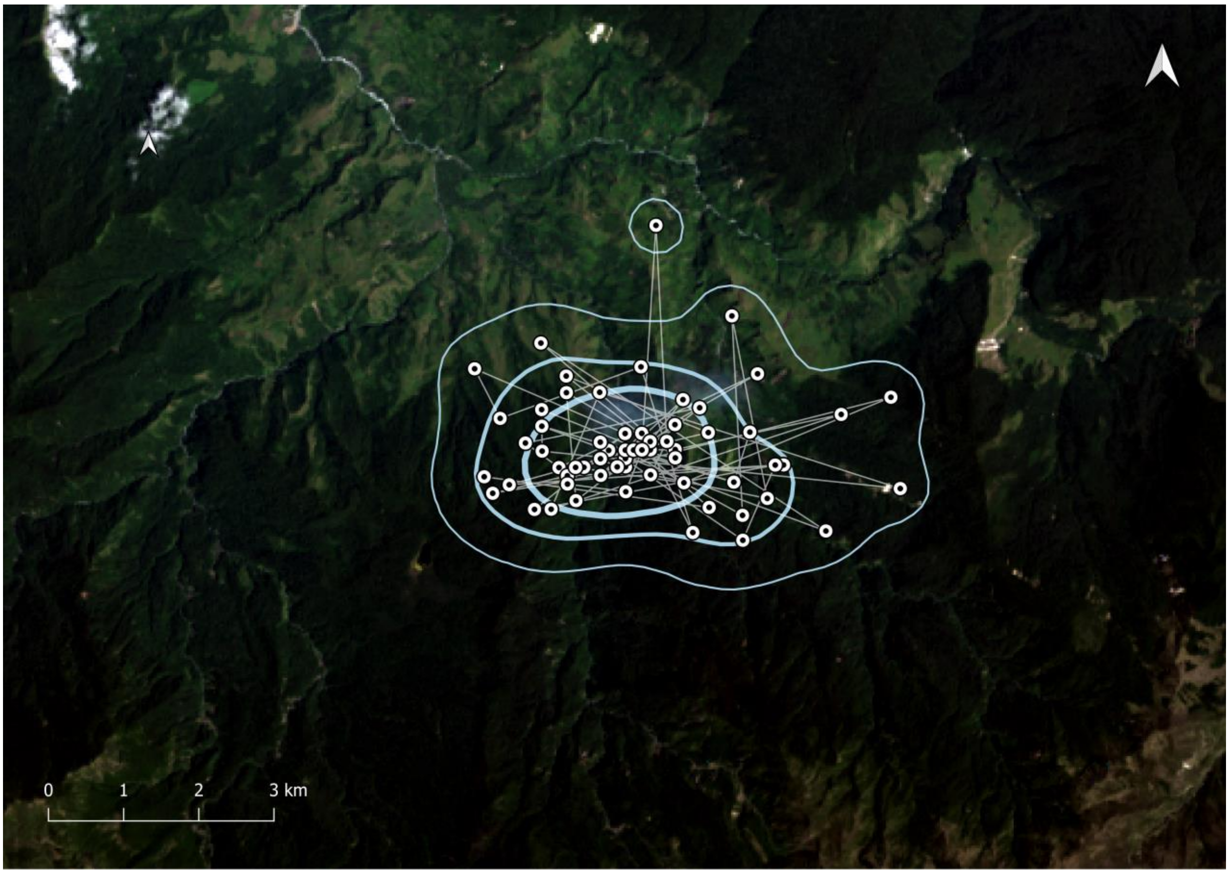

S15 Fig. Huon Bowerbird 36515 year 1 tracking data (8 Nov 2018 – 7 Nov 2019).

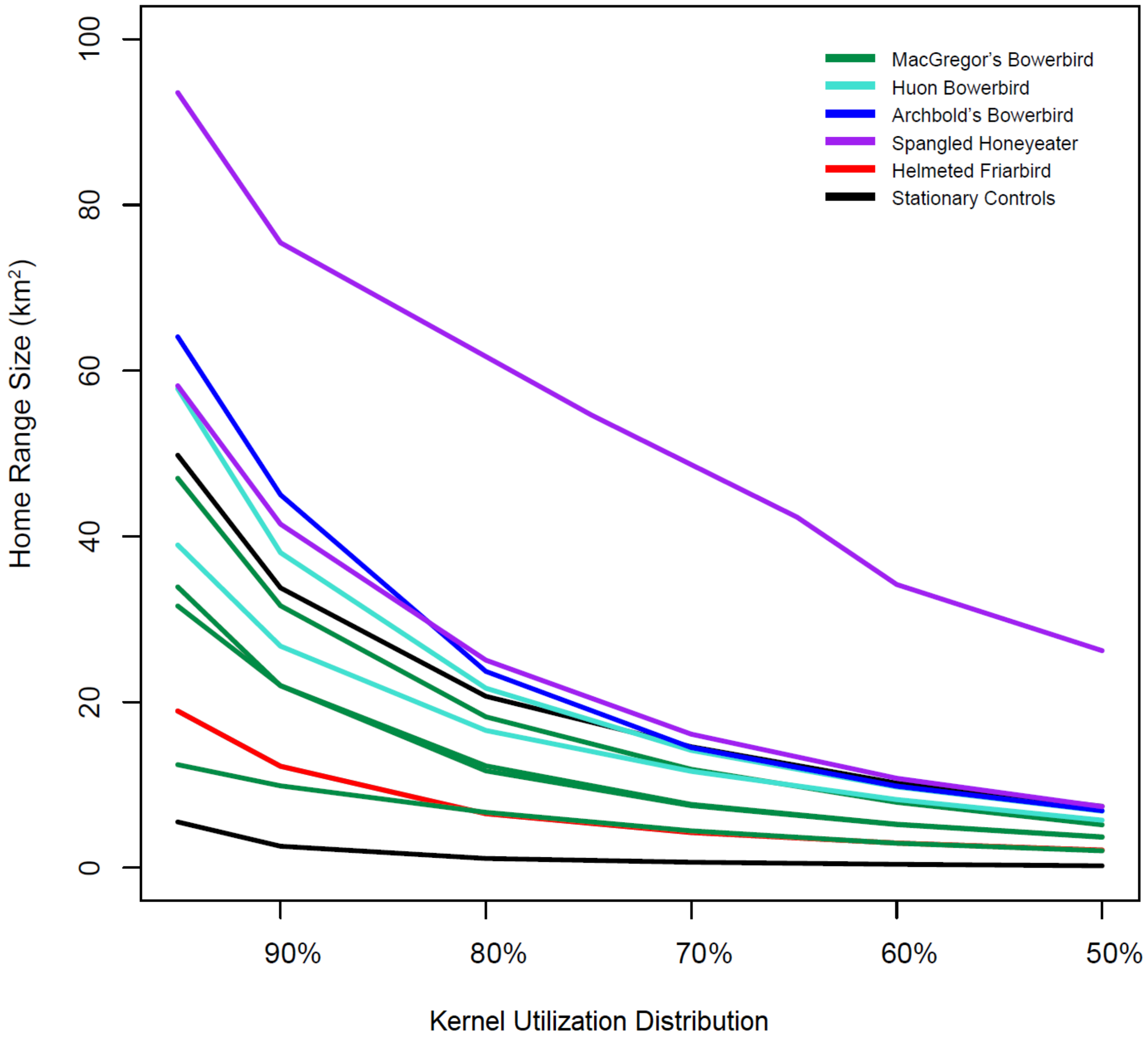

S16 Fig. Home range size estimates as in Fig 4, color-coded by species.

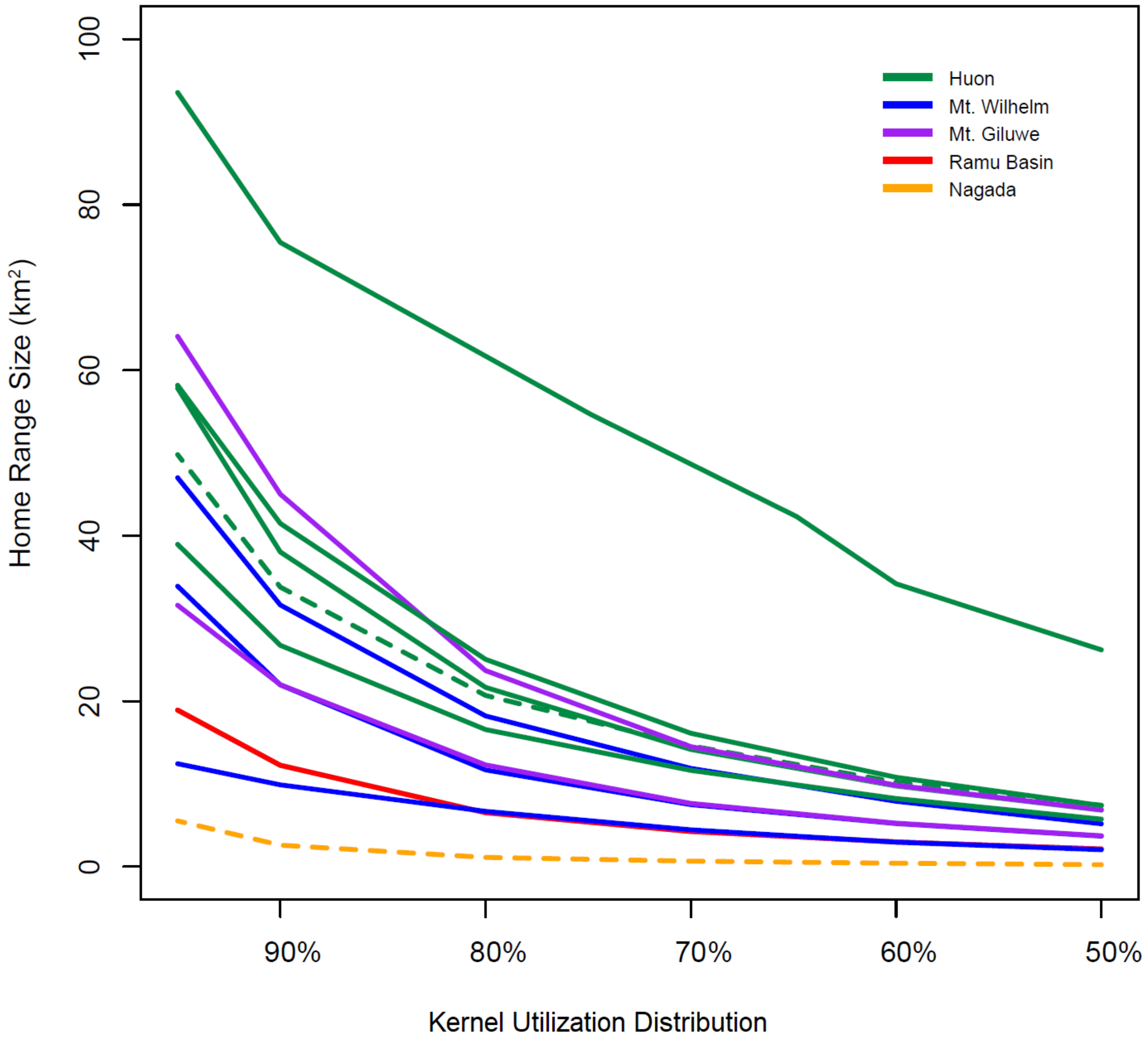

S17 Fig. Home range size estimates as in Fig 4, color-coded by study area. Dotted lines indicate stationary control transmitters.

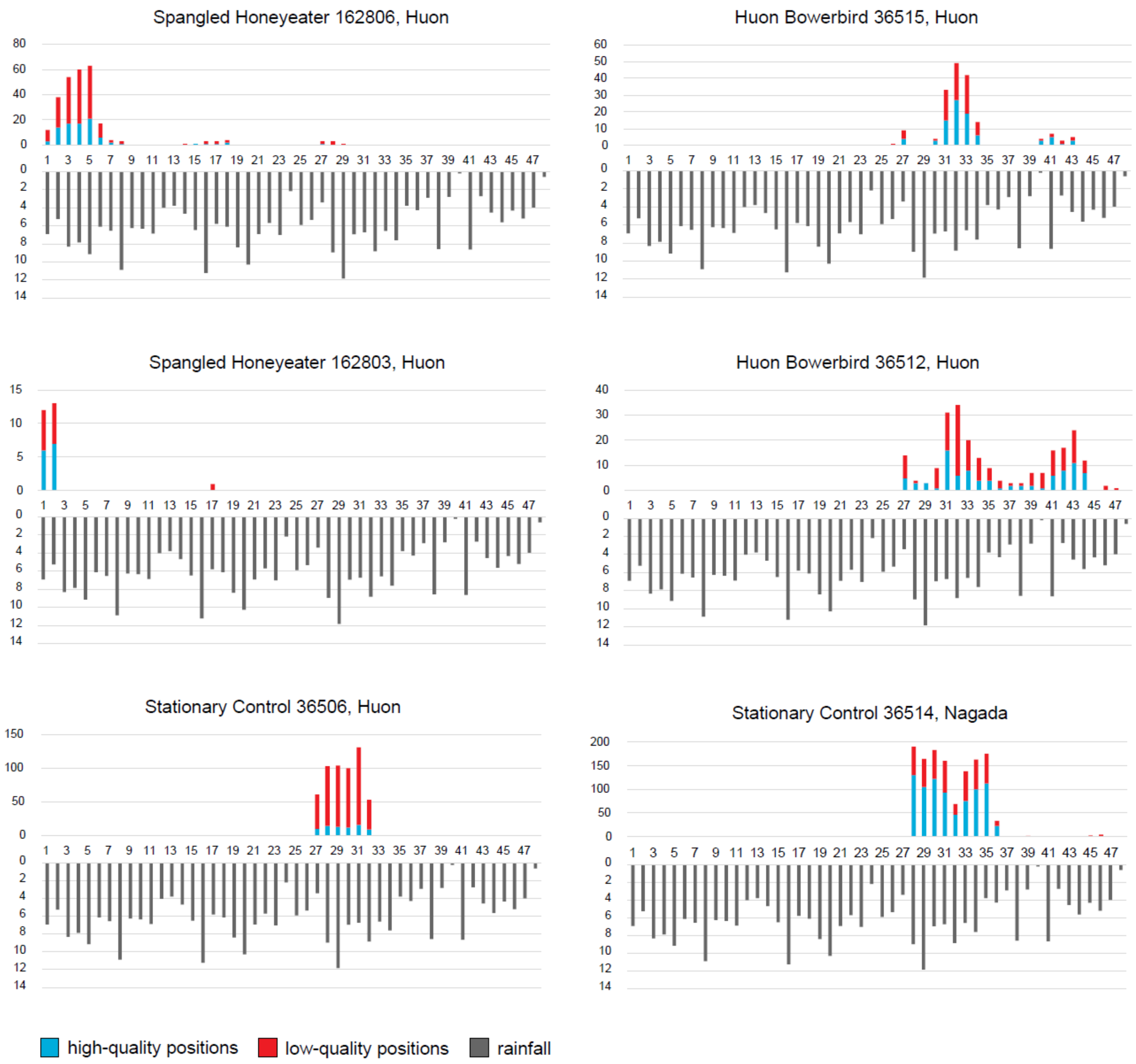

S18A Fig. Birds from Huon; stationary control transmitters from Huon and Nagada.

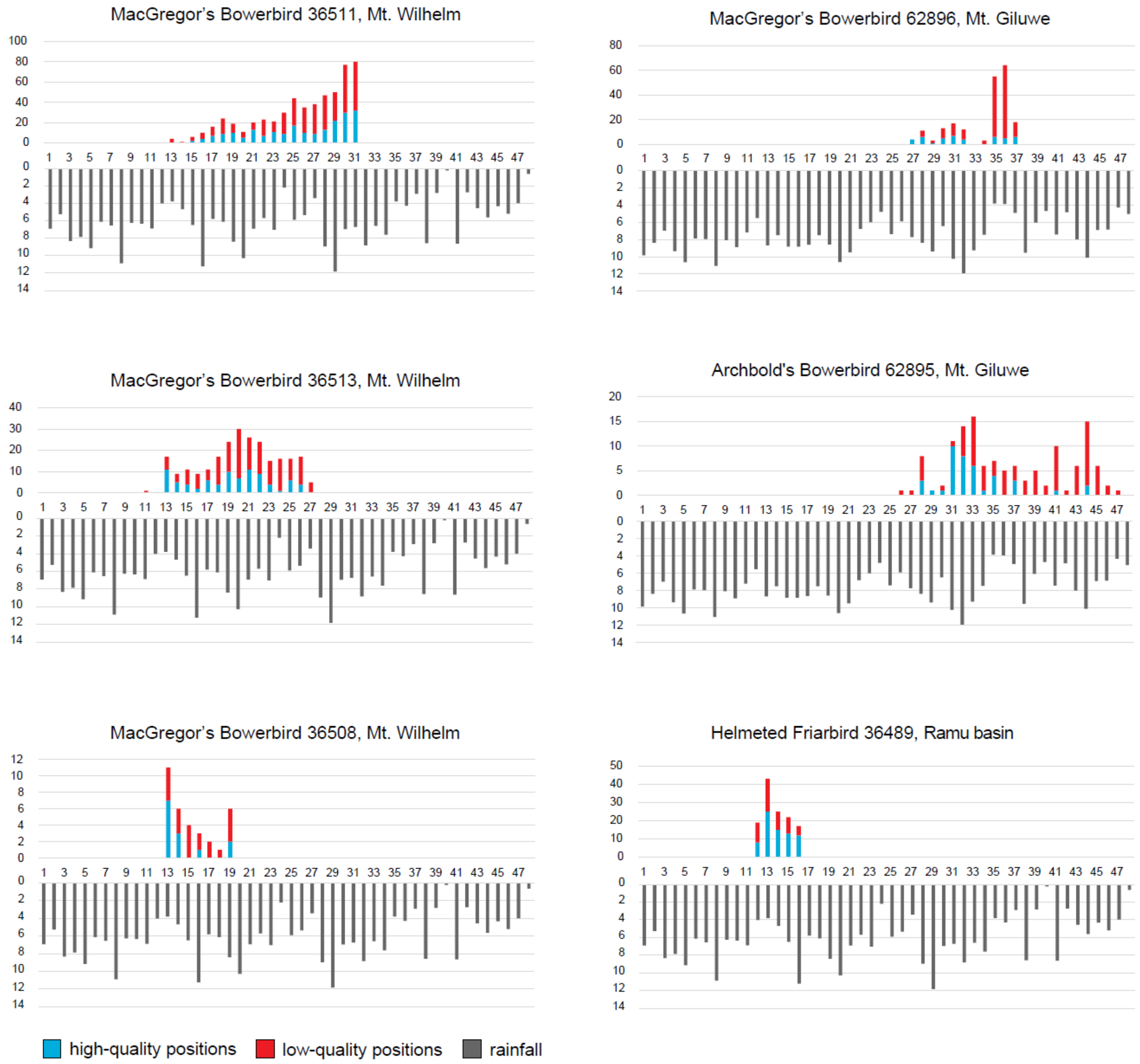

S18B Fig. Birds from Mt. Wilhelm, Mt. Giluwe, and the Ramu basin.

S1 Table. Genetic sex information for tracked birds. We only sexed birds with > 10 high-quality positions (Argos classes 1, 2, and 3); the three individuals with < 10 high-quality positions are not listed here. Individuals without blood samples were not genetically sexed; these include two bowerbirds identified as adult males based on plumage characters. Blood samples are deposited at the Natural History Museum of Denmark.

Species	Transmitter	Blood Museum Code	Sex
MacGregor’s Bowerbird	36511	ZMUC 615743	female
MacGregor’s Bowerbird	36513	ZMUC 615815	female
MacGregor’s Bowerbird	36508	ZMUC 615816	male
MacGregor’s Bowerbird	62896	NA	presumed male
Huon Bowerbird	36512	ZMUC 615947	female
Huon Bowerbird	36515	ZMUC 615948	female
Archbold's Bowerbird	62895	NA	presumed male
Spangled Honeyeater	162803	NA	unknown
Spangled Honeyeater	162806	NA	unknown
Helmeted Friarbird	36489	ZMUC 615638	male
